# Alternative Sigma Factor Over-Expression Enables Heterologous Expression of a Type II Polyketide Biosynthetic Pathway in *Escherichia coli*

**DOI:** 10.1371/journal.pone.0064858

**Published:** 2013-05-28

**Authors:** David Cole Stevens, Kyle R. Conway, Nelson Pearce, Luis Roberto Villegas-Peñaranda, Anthony G. Garza, Christopher N. Boddy

**Affiliations:** 1 Department of Chemistry, University of Ottawa, Ottawa, Ontario, Canada; 2 Department of Biology, Syracuse University, Syracuse, New York, United States of America; 3 Department of Biology, University of Ottawa, Ottawa, Ontario, Canada; Laurentian University, Canada

## Abstract

**Background:**

Heterologous expression of bacterial biosynthetic gene clusters is currently an indispensable tool for characterizing biosynthetic pathways. Development of an effective, general heterologous expression system that can be applied to bioprospecting from metagenomic DNA will enable the discovery of a wealth of new natural products.

**Methodology:**

We have developed a new *Escherichia coli*-based heterologous expression system for polyketide biosynthetic gene clusters. We have demonstrated the over-expression of the alternative sigma factor σ^54^ directly and positively regulates heterologous expression of the oxytetracycline biosynthetic gene cluster in *E. coli*. Bioinformatics analysis indicates that σ^54^ promoters are present in nearly 70% of polyketide and non-ribosomal peptide biosynthetic pathways.

**Conclusions:**

We have demonstrated a new mechanism for heterologous expression of the oxytetracycline polyketide biosynthetic pathway, where high-level pleiotropic sigma factors from the heterologous host directly and positively regulate transcription of the non-native biosynthetic gene cluster. Our bioinformatics analysis is consistent with the hypothesis that heterologous expression mediated by the alternative sigma factor σ^54^ may be a viable method for the production of additional polyketide products.

## Introduction

Bacterial polyketides possess an enormous range of chemical diversity and biological function. Many polyketides such as tetracycline [1], epothilone [2], and rapamycin [3] have been developed into key clinical pharmaceuticals in a broad range of therapeutic areas [4]. Sequencing of bacterial genomes, especially those of major polyketide producers such as *Actinomycetes* and δ-*proteobacteria*, have shown that there are many more polyketide biosynthetic pathways than polyketides isolated from standard cultivation techniques [5]–[7]. These genetically encoded polyketide natural products from cultivatable and uncultivatable bacteria represent one of the greatest remaining untapped reservoirs of new natural product diversity. Methods to effectively access this diversity will have a major impact on drug discovery [8].

To access this untapped diversity of polyketide products, a general method for heterologous expression of these pathways is needed. The selection of a heterologous host is contingent upon its ability to efficiently transcribe non-native pathways, translate the often GC rich transcripts, and possess all the starter and extender units necessary for polyketide production [9]. Hosts highly related to the native polyketide-producing organism often meet these requirements. Hosts such as *Streptomyces coelicolor* [10], *Streptomyces lividans* [11], and *Myxococcus xanthus* [12] have proved successful for heterologous production of a number of different polyketide products. Screening of multiple hosts related to the producing organism is often necessary to identify a successful heterologous host [13], [14]. In addition, various culture conditions also need to be screened to identify conditions that produce the desired compounds [15]. The lack of a general heterologous expression system has made heterologous expression incompatible with screening bacterial genomic DNA libraries for new polyketide products.

*Escherichia coli* has a number of advantages that make it an appealing host for a heterologous expression system. The ease of genetic manipulation and culturing as compared to other heterologous hosts makes *E. coli* far more useful for bioprospecting from genomic DNA libraries. Codon usage as well as starter and extender unit availability have proven not to be obstacles for heterologous production in *E. coli* [16]–[18]. The principle impediment to the use of *E. coli* as a heterologous host is its inability to effectively transcribe heterologous pathways. In all examples of heterologous expression of polyketides in *E. coli*, all of the biosynthetic genes have been placed under the control of the T7 promoter [16], [17]. The native promoters present in these heterologous pathways are not sufficient to ensure expression of all the genes in a given pathway under standard *E. coli* culture conditions.

To convert *E. coli* into a general heterologous expression system, a mechanism for ensuring transcription of all the biosynthetic genes in a foreign pathway is required. We hypothesized that a general transcriptional regulator of polyketide biosynthesis should be present in bacteria because horizontal transfer of biosynthetic pathways is a key mechanism for their proliferation among species [19]. Strong evidence for substantial horizontal transfer of pathways can be seen in the large number of diverse *Streptomyces* strains which produce the tetracycline family of antibiotics, the presence of yersiniabactin-like gene clusters in diverse *Actinomycetes* [6], and the presence of highly related pederin, onnamide A and psymberin gene clusters in unidentified bacterial strains from the beetle *Paederus fuscipes* and the sponges *Theonella swinhoei* and *Psammocinia* aff. *bulbosa* respectively [20]. Because polyketide production is positively regulated in most bacteria by stress [21], we further hypothesized that alternative sigma factors downstream of the stringent response should positively regulate transcription of biosynthetic genes.

Herein we show that this new mechanism for heterologous expression of the oxytetracycline polyketide biosynthetic pathway, where a high-level pleiotropic sigma factor from the heterologous host is used to positively regulate transcription of the non-native biosynthetic gene cluster, is highly effective. We demonstrate that one of the six alternative sigma factors in *E. coli* can selectively, directly, and positively regulate heterologous expression from the *Streptomyces rimosus* oxytetracycline biosynthetic gene cluster [1] in *E. coli*. This is the first successful heterologous production of an aromatic polyketide in *E. coli* [22], demonstrating the utility of this method.

## Results

### Standard *E. coli* culture conditions do not enable heterologous expression

**Figure 1:**
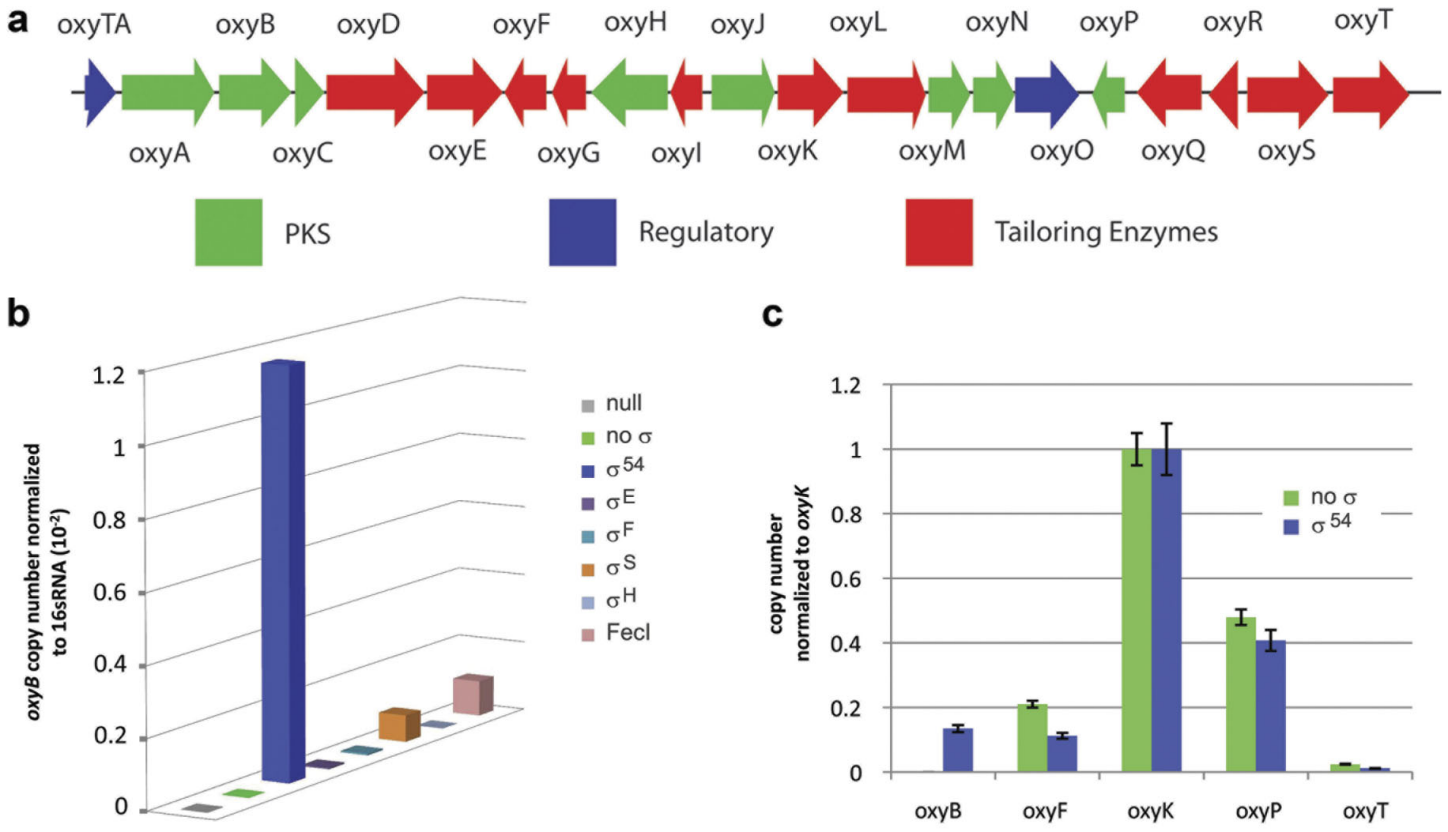
SYBR-green based qPCR analysis shows that transcription limits heterologous production of oxytetracycline in *E. coli*. (**a**) The 32 kb oxytetracycline biosynthetic gene cluster is shown. Five putative operons, *oxyABCDE*, *oxyIHGF*, *oxyJKLMNO*, *oxyRQP*, and *oxyST* are predicted for this gene cluster (**b**) qPCR analysis shows that over-expression of the alternative sigma factors σ^54^, σ^S^ and FecI enable detectable levels of the *oxyB* transcript to be produced. Over-expression of no sigma factor, σ^E^, σ^F^ and σ^H^ do not lead to detectable levels of the *oxyB* transcript. (**c**) qPCR analysis shows that over-expression of the alternative sigma factor σ^54^ lead to detectable levels of transcripts for all five putative operons in the oxytetracycline biosynthetic pathway. In the absence of σ^54^ over-expression, the *oxyB* transcript cannot be detected. See also Table S1. No *oxyB, oxyF, oxyK, oxyP, oxyT* transcripts were observed in the null strains which lacked the oxytetracycline gene cluster. (Figures S1, S2).

To test our hypothesis that an alternative sigma factor downstream of the stringent response could positively regulate transcription of polyketide biosynthetic pathways in *E. coli*, we investigated the ability of *E. coli* to heterologously express the oxytetracycline biosynthetic pathway from *S. rimosus* [1]. Oxytetracycline is produced by a 32-kb type II polyketide synthase gene cluster consisting of 21 genes. Thirteen of these genes encode proteins required for the biosynthesis of oxytetracycline [23] and thus must be transcribed for successful heterologous production of oxytetracycline. The small intergenic spaces between open reading frames and the changes in direction of transcription in the oxytetracycline gene cluster suggest that the entire pathway is expressed as, at minimum, five putative operons with at least one biosynthetic gene on each transcript (Figure 1a).

To determine if *E. coli* could produce oxytetracycline under standard culture conditions, *E. coli* BAP1 [16] was transformed with the *S. rimosus* oxytetracycline pathway [12] and cultured in rich liquid media. BAP1 was used because it possesses a chromosomal insertion of the phosphopantetheinyl transferase from *Bacillus subtillis*, *sfp*, which ensures that polyketide synthase acyl carrier proteins (ACPs) are post-translationally modified [16]. LC-MS/MS analysis of the organic extracts from the culture media did not show any detectable oxytetracycline in the culture broth (see Figure S3). LC-MS/MS analysis was performed using multiple reaction monitoring (MRM) mode with a lower limit of detection of 100 µg/L. These results indicate that *E. coli*, under standard culture conditions, cannot heterologously produce oxytetracycline from the native *S. rimosus* gene cluster.

To determine if transcription of the genes in the oxytetracycline gene cluster was limiting production of oxytetracycline in *E. coli*, we quantified mRNA levels using quantitative PCR (qPCR) for five genes in the pathway. The five genes *oxyB*, *oxyF*, *oxyK*, *oxyP*, and *oxyT*, one per putative operon, were selected. *oxyB* encodes the β-ketosynthase, *oxyF* and *oxyT* encode methyltransferases and *oxyK* encodes an aromatase all of which are required for oxytetracycline biosynthesis [23]. *oxyP* encodes a malonyl-ACP transferase, which may not be required for biosynthesis of oxytetracycline [23]. Transcripts for four of the genes, *oxyF*, *oxyK*, *oxyP* and *oxyT* could be easily detected and quantified (Figure 1c). Transcript levels for *oxyB* were indistinguishable from negative control samples that lacked the oxytetracycline gene cluster, indicating that *oxyB* was not transcribed (Figure 1b, Figure S1, Table S1). As OxyB is required for generation of the polyketide backbone of oxytetracycline, the lack of transcription of this gene is sufficient to account for the inability of *E. coli* to produce oxytetracycline under standard culture conditions. These results, in conjunction with published work [16], [17], confirm that transcription of heterologous polyketide pathways in *E. coli* limits the ability of the heterologous host to produce polyketide products when using the native promoters.

### Over-expression of alternative sigma factors σ^54^, σ^S^, and FecI positively regulate transcription of *oxyB* in *E. coli*

To test our hypothesis that an alternative sigma factor downstream of the stringent response could positively regulate transcription of polyketide biosynthetic pathways in *E. coli*, we investigated the impact of alternative sigma factor over-expression on transcription from the oxytetracycline gene cluster. In their native hosts, polyketide biosynthetic gene clusters are principally regulated by one or more pathway specific regulators, which can respond to a diversity of stimuli [21], [24]. The most general observed positive regulator of polyketide biosynthesis is nutrient limitation [21], which initiates the stringent response in bacteria. During stringent response the levels of the pleiotropic regulators phosphorylated guanosine nucleotides, ppGpp and pppGpp, increase, leading to an increase in alternative sigma factor-mediated transcription [24]. As increases in (p)ppGpp have been shown to correlate with polyketide production in diverse bacterial species [25], [26], we hypothesized that this positive regulation could be mediated by alternative sigma factors.

Sigma factors control the specificity of gene transcription by binding to RNA polymerase (RNAP) and recruiting it to promoter sequences upstream of the gene to be transcribed [27]. In addition to the household sigma factor σ^70^, *E. coli* has six alternative sigma factors, σ^54^ which is involved in nitrogen assimilation, σ^H^ which controls heat shock promoters, σ^S^ which controls stationary phase promoters, σ^F^ which controls flagellum related genes, σ^E^ controls response to extracytoplasmic stress, and FecI which is involved in iron transport [27]. Over-expression of sigma factors has been shown to increase expression of genes in their regulon in many bacteria, including *E. coli* [28]–[30]. If an alternative sigma factor is a positive regulator of polyketide biosynthesis, over-expression of that sigma factor will increase transcription from genes in the oxytetracycline gene cluster.

To determine if σ^54^,σ^H^, σ^S^, σ^F^, σ^E^, and FecI could positively regulate polyketide biosynthesis in *E. coli*, we quantified mRNA levels using quantitative PCR for *oxyB*. Six *E. coli* BAP1 strains possessing the oxytetracycline biosynthetic gene cluster and over-expressing either σ^54^,σ^H^, σ^S^, σ^F^, σ^E^, or FecI were investigated. No *oxyB* transcripts could be detected when σ^H^, σ^F^, and σ^E^ were over-expressed. Over-expression of σ^S^ and FecI led to detectable but low levels of *oxyB* transcription. When σ^54^ was over-expressed substantial levels of *oxyB* transcripts could be detected (Figure 1b, Figure S2, Table S1). Quantification of mRNA levels for *oxyB*, *oxyF*, *oxyK*, *oxyP* and *oxyT* in *E. coli* BAP1 possessing the oxytetracycline biosynthetic gene cluster and over-expressing *E. coli* σ^54^ showed that we were able to detect all five transcripts (Figure 1c). These data demonstrate that all of the putative transcripts required for oxytetracycline are present when σ^54^ is over-expressed.

### Over-expression of σ^54^ enables oxytetracycline production in *E. coli*

**Figure 2:**
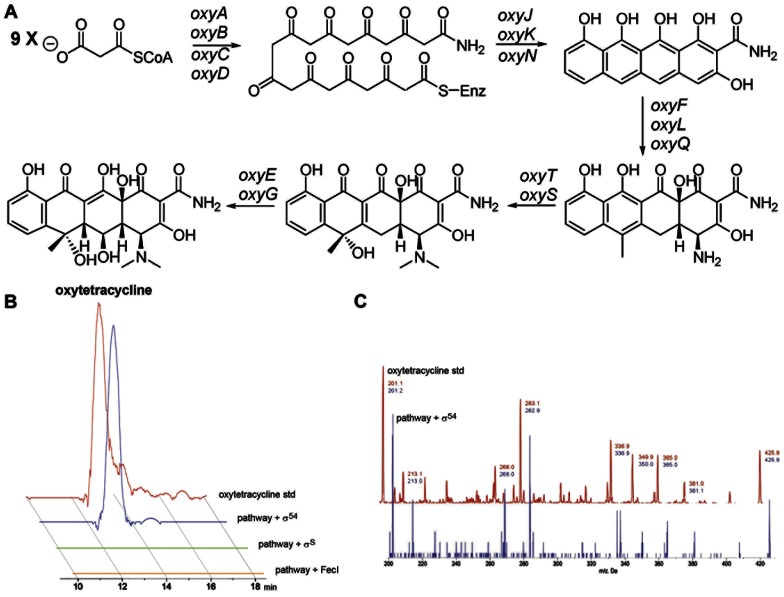
Over-expression of σ^54^ enables *E. coli* to heterologously produce oxytetracycline. (**a**) The enzymatic pathway responsible for the biosynthesis of oxytetracycline. (**b**). ESI-LC-MS/MS analysis of an oxytetracycline standard and the organic extracts from *E. coli* cultures containing the oxytetracycline gene cluster and over-expressing σ^54^, σ^S^, or FecI. These traces show the ion extraction data from the Q3 scan of MS/MS experiments. Q1 was used to select the [MH]^+^ ion for oxytetracycline (*m*/*z* = 461). Ion extractions were performed for the oxytetracycline fragment *m*/*z* = 283 from the Q3 scan. Signals with a peak width of less than 0.1 s were regarded as noise and removed with the noise filter application. (**c**). The MS^2^ spectrum of the m/z = 461 peak for the oxytetracycline authentic standard and heterologously produced oxytetracycline. See also Figure S3.

If transcription limits heterologous production of polyketides in *E. coli*, then over-expression of σ^54^ in the presence of the oxytetracycline gene cluster is predicted to lead to the production of oxytetracycline. To test this hypothesis we over-expressed σ^54^ in *E. coli* BAP1 containing the oxytetracycline gene cluster and analyzed the organic extracts with LC-MS/MS to determine if oxytetracycline was detectable. LC-MS/MS analysis using the MS^2^ scan mode clearly identified the presence of oxytetracycline (Figure 2b). The LC retention time and MS^2^ spectrum of our heterologously produced oxytetracycline was indistinguishable from authentic oxytetracycline standards (Figure 2c). Using MRM mode and a standard curve generated from authentic oxytetracycline, the titer of heterologously expressed oxytetracycline was determined to be 2.0±0.1 mg per L of culture broth. These results demonstrate that when σ^54^ is over-expressed in *E. coli* containing the oxytetracycline gene cluster, all the biosynthetic genes are transcribed and translated, all the necessary proteins are functional *in vivo*, and the required starter and extender units are available, enabling oxytetracycline production.

Because over-expression of σ^S^ and FecI also produced detectable *oxyB* transcripts, it is possible that these alternative sigma factors may also enable heterologous production of oxytetracycline. We therefore investigated the ability of *E. coli* BAP1 cultures over-expressing σ^S^ and FecI to heterologously produce oxytetracycline. LC-MS/MS analysis showed that no detectable oxytetracycline was present in these culture broths (Figure 2b). As expected from our transcription data, over-expression of σ^H^, σ^F^, and σ^E^ did not lead to heterologous production of detectable levels of oxytetracycline (Figure S3). These data demonstrate that only the alternative sigma factor, σ^54^, can positively regulate transcription of the oxytetracycline biosynthetic pathway, leading to heterologous production of oxytetracycline in *E. coli*.

### Antibiotic activity of oxytetracycline does not limit heterologous expression

Oxytetracycline, like tetracycline, is a broad spectrum antibiotic that binds to the 30S subunit of the ribosome, inhibiting protein synthesis [31]. The minimum inhibitor concentration of *E. coli* strains to oxytetracycline can range from 0.5–16 µg/mL with a typical value of 8 µg/mL [32]. Because the oxytetracycline gene cluster used in this study lacked a resistance gene, we were concerned that the activity of oxytetracycline at our maximum titer of 2 ug/mL may limit heterologous expression. To address this concern, we generated a tetracycline resistance vector pLRVP09 based on pBR322 and transformed this into a BAP1 strain with the oxytetracycline gene cluster and a σ^54^ expression vector. Heterologous production of oxytetracycline from this strain was identical to strains lacking the pBR322 derived TetC resistance determinant. This data indicates that the antibiotic activity of oxytetracycline does not limit the heterologous production of oxytetracycline to 2 mg/L.

### σ^54^ over-expression does not lead to substantial accumulation of the oxytetracycline biosynthetic proteins

While most classes of bacterial natural products have been heterologously expressed in *E. coli*, type II polyketide biosynthetic pathways have not been [22]. Even when key type II biosynthetic genes, such as those encoding the ketosynthase (KS) or chain length factor (CLF), are placed under the control of known *E. coli* promoters, it has not been possible to produce type II polyketides, due predominantly to the production of insoluble KS-CLF heterodimer [22], [33]. Because σ^54^ over-expression leads to production of oxytetracycline, some soluble, functional KS (OxyA) and CLF (OxyB) must be expressed. To evaluate if σ^54^ over-expression constituted an effective method to produce soluble KS-CLF heterodimer, we examined the soluble fraction of oxytetracycline producing strains by SDS-PAGE (Figure S4). While a band consistent with σ^54^ (54 kDa) could be easily detected, there was no evidence for high-level production of OxyA (45 kDa) or OxyB (44 kDa). Thus while σ^54^ over-expression must lead to some functional soluble KS-CLF heterodimer, there is not substantial accumulation of these key proteins.

### σ^54^ directly regulates heterologous production in *E. coli*

**Figure 3:**
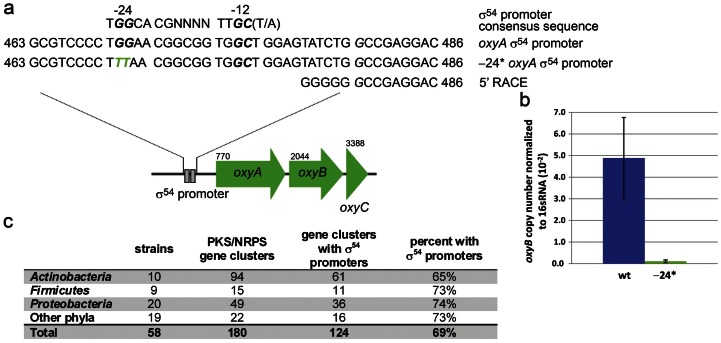
Putative σ^54^ promoters are found in the majority of polyketide and non-ribosomal peptide biosynthetic gene clusters. (**a**) The *oxyABCDEF* operon from the *S. rimosus* oxytetracycline biosynthetic pathways contains a putative σ^54^ promoter. The promoter shows high homology to the σ^54^ promoter consensus sequence especially at the key −12 and −24 positions. A mutation of the highly conserved GG residues at −24 (−24*) and 5′ RACE were used to confirm that this promoter is responsible for direct, positive transcriptional regulation of the *oxyB* gene by σ^54^ over-expression. (**b**) Transcription of the *oxyABCDEF* operon is directly controlled by the σ^54^ promoter upstream of *oxyA*. Mutation of the highly conserved GG residues −24 from the transcriptional start site to TT leads to a greater than 40 fold decrease in *oxyB* transcript levels as determined by qPCR. qPCR data was obtained from BAP1 transformed with pDCS11 and either pDCS61 or pDCS62. (**c**) Results of a bioinformatics analysis of 58 bacterial genomes containing 180 polyketide synthase (PKS) and non-ribosomal peptide synthetase (NRPS) biosynthetic gene clusters show that the majority of gene clusters contain putative σ^54^ promoters. It is particularly intriguing that *Actinobacteria* possess gene clusters with σ^54^ promoters since they lack the gene encoding σ^54^. See also Tables S2, S3, S4.

σ^54^ can positively regulate transcription directly or indirectly. For direct regulation, the σ^54^-RNAP complex binds to a σ^54^ consensus promoter upstream of the operon under σ^54^ control. The σ^54^ consensus promoter is unique among bacterial promoters with highly conserved residues −12 and −24 from the transcriptional start site (T*GG*CACG-N4-TT*GC*(T/A)) [34] and can reliably be identified from sequence data. If σ^54^ is directly regulating transcription of *oxyB*, a σ^54^ promoter consensus sequence must be present upstream of the operon containing *oxyB*. To determine if σ^54^ promoters were present in the oxytetracycline biosynthetic pathway the entire gene cluster was examined for σ^54^ promoter consensus sequences using the web-based tool PromScan (http://molbiol-tools.ca/promscan/) [35]. A high scoring consensus promoter was located upstream of the operon containing *oxyB* in a location that would allow direct transcription of the putative operon (Figure 3a, Table S2). The location and consensus sequence of the identified promoter provides strong evidence for direct regulation by σ^54^ of the heterologous production of oxytetracycline in *E. coli*.

To demonstrate that the predicted σ^54^ promoter is functional and involved in the transcriptional regulation of *oxyB*, the highly conserved GG −24 from the transcriptional start site was mutated to TT. The −24 region plays a major role in anchoring the σ^54^-RNAP complex to the promoter and mutation of the conserved GG is known to decrease transcription of genes under σ^54^ control by ten to one hundred-fold [36], [37]. qPCR analysis showed that *oxyB* transcript levels from the TT mutant σ^54^ promoter were greater than forty-fold lower as compared to the wild type (Figure 3b, Table S1). To identify promoter elements involved in transcription of the operon containing *oxyB*, we performed 5′ Rapid Amplification of cDNA Ends (5′ RACE). Our 5′ RACE data identified a transcriptional start site consistent with the proposed σ^54^ promoter, confirming that this promoter is functional and that *oxyB* transcription is directly regulated by σ^54^.

### σ^54^ promoters are common in polyketide and non-ribosomal peptide biosynthetic pathways

If σ^54^ promoters are commonly found in polyketide and non-ribosomal peptide biosynthetic pathways, σ^54^ over-expression may be a general route to heterologous expression of polyketides and non-ribosomal peptides. To evaluate this possibility, we examined the genomes of 58 sequenced bacteria for polyketide and non-ribosomal peptide biosynthetic gene clusters and σ^54^ promoters. The 58 species included major secondary metabolite producers such as 10 *Actinobacteria*, 20 *Proteobacteria*, 9 *Firmicutes*, as well as 19 species from diverse phyla. This selection of species contains representative examples of most of the sequenced bacterial phylogenic diversity.

Manual examination of all genes annotated as polyketide synthase, 3-oxoacyl acyl carrier protein synthase, and non-ribosomal peptide synthetase (COG3321, COG0304, and COG1020 respectively) [38] and their adjacent genes, identified 180 polyketide and non-ribosomal peptide biosynthetic gene clusters (Figure 3c, Table S3).

Each bacterial genome was analyzed to identify all putative σ^54^ promoters. To identify σ^54^ promoters, a positional weighted matrix (PWM) describing the σ^54^ promoter consensus sequence [36] was aligned with all sites on the forward and reverse strands of the genome. The fit at each position was scored using the published algorithm [35] from the bioinformatics tool PromScan. Sequences with a fit scoring 75 or greater and on the same strand, less than 500 bases upstream of a start codon were identified as putative σ^54^ promoters. All experimentally validated σ^54^ promoters in the *E. coli* genome had scores ranging from the 80 s to high 90 s [30]. In GC rich organisms such as *M. xanthus* a number of previously identified σ^54^ promoters had scores in the low to mid 70 s [39], [40]. Based on these data, a score of 75 is expected to provide the required low level of false negatives across a broad range of genomes.

To evaluate the level of false positives, a sequence randomized *E. coli* genome was analyzed [41]. 25.6% of genes in the native *E. coli* genome and 18.4% of the genes in the sequence randomized genome were predicted to have σ^54^ promoters. 18.4% thus provides a baseline for the level false positive predictions. This high false positive rate is typical of PWM based promoter prediction tools [42], [43].

As false positives are not expected to be conserved across species, a comparative genomic approach that examines the predicted promoters across a wide range of bacterial genomes should decrease the false positive rate and increases the specificity [44]. Our data shows that >50% of the bacterial genomes possessing a *glnA* ortholog (COG0174), the archetypical gene under direct σ^54^ transcriptional control, had one or more predicted σ^54^ promoters upstream of this gene. In comparison only 10% of the genomes examined had predicted σ^54^ promoters upstream of the well-characterized non-σ^54^ regulated ribosomal proteins encoding genes (COG0093, COG0049) [45], transcription elongation factor encoding genes (COG0264, COG0231) [46], [47], and the ATP synthase encoding genes (COG005, COG0224) [48]. Thus while individual promoter predictions have a high false positive rate, correlating promoter predictions across multiple species for individual orthologs substantially decreases the false positive predictions and increases the ability to accurately predict orthologs with σ^54^ promoters.

Of the 180 polyketide and non-ribosomal peptide biosynthetic gene clusters identified, 124 (69%) contained one or more σ^54^ promoters appropriately positioned to regulate transcription (Table S3, Table S4). 24 gene clusters possessed a single σ^54^ promoter, however the vast majority had two or more, with some pathways possessing up to two dozen σ^54^ promoters. Additionally 75 of the 124 (60%) clusters had a putative σ^54^ promoter appropriately placed to directly regulate either a polyketide synthase or a non-ribosomal peptide synthetase encoding ortholog. These results demonstrate that a majority (69%) of polyketide and non-ribosomal peptide biosynthetic gene clusters possess putative σ^54^ promoters and that these promoters are appropriately positioned to regulate transcription of at least one operon in these gene clusters. These results are consistent with the hypothesis that σ^54^ over-expression may be a general method for ensuring transcription of polyketide and non-ribosomal peptide biosynthetic gene clusters in heterologous hosts, such as *E. coli*.

## Discussion

Heterologous expression of polyketide and non-ribosomal peptide biosynthetic gene clusters will play a major role in the discovery of new natural products from metagenomic and environmental DNA samples. Currently no general heterologous expression method appropriate for screening DNA libraries exists. Herein we describe a new mechanism for heterologous expression of a polyketide biosynthetic pathway, where a high-level, pleiotropic alternative sigma factor from the heterologous host positively regulates transcription of the biosynthetic gene cluster. In contrast, known methods for heterologous expression rely on either replacing each native promoter with known, well-characterized promoter from the heterologous host, such as the T7 promoter in *E. coli* [16]–[18], or rely on the heterologous host to constitutively express each gene from the native promoters [10]–[15]. Our approach, which actively induces transcription of gene clusters by over-expression of alternative sigma factors, may provide a general solution to the heterologous expression problem that is compatible with screening DNA libraries.

Our results demonstrate that over-expression of the sigma factor, σ^54^, enables efficient heterologous expression of oxytetracycline biosynthetic gene cluster in *E. coli*. Using qPCR, we have demonstrated that no transcript is detectable under standard culture conditions for the key oxytetracycline biosynthetic gene *oxyB*. LC-MS/MS analysis of these cultures showed no detectable oxytetracycline production in the absence of detectable *oxyB* transcripts. Over-expression of σ^54^ positively regulates transcription of *oxyB*, generating detectable levels of *oxyB* transcripts, enabling production of oxytetracycline. Bioinformatics analysis of the oxytetracycline biosynthetic gene cluster revealed a σ^54^ promoter sequence appropriately positioned to directly regulate *oxyB* transcription. Site directed mutagenesis and 5′ RACE strongly support that this promoter is functional. Further bioinformatic analysis of 180 polyketide and non-ribosomal peptide biosynthetic pathways shows that the majority of these pathways possess putative σ^54^ promoter sequences (69%), suggesting that σ^54^-mediated heterologous expression may be a general phenomenon.

Substantial evidence exists to support the hypothesis that over-expression of alternative sigma factors, such as σ^54^, can positively regulate diverse polyketide biosynthetic pathways. Nutrient limitation, which is one of the only known general regulators of polyketide biosynthesis [21], initiates the stringent response in bacteria. During stringent response the levels of the global bacterial regulator (p)ppGpp increases. Increases in (p)ppGpp have been shown to correlate with polyketide production in diverse bacterial species [25], [26]. For example *relA*, the gene encoding the (p)ppGpp synthetase, in *S. coelicolor* is required to trigger polyketide biosynthesis^26^. (p)ppGpp disrupts the interaction between the RNA polymerase (RNAP) and the principle sigma factor, σ^70^, enabling RNAP to interact with alternative sigma factors, which increases alternative sigma factor-mediated transcription [24]. Thus nutritional limitation and (p)ppGpp exert their regulatory effects by increasing alternative sigma factor-mediated transcription, suggesting that polyketide biosynthetic pathways may be under either direct or indirect alternative sigma factor transcriptional control.

We investigated the effect of over-expression of all the alternative sigma factors from *E. coli* on the transcription of *oxyB* and on the production of oxytetracycline. Only σ^54^ over-expression led to both detectable levels of the *oxyB* transcript and detectable levels of oxytetracycline production. σ^54^ is known to play a major role in response to nitrogen limitation in most bacteria [27] and sporulation in *delta-proteobacteria* [49]. Interestingly, both nitrogen limitation and sporulation correlate with polyketide production [21], [25]. While this suggests that σ^54^ may play a role in regulation of polyketide biosynthesis in some bacteria, substantial work in native hosts is required to test this hypothesis.

A unique aspect of σ^54^-mediated transcription is that it requires co-activation to initiate transcription [50]. Enhancer binding proteins (EBPs) allow σ^54^-loaded RNA polymerase to form a transcriptionally active open complex. EBPs contain a DNA binding domain that interacts with a specific DNA sequence called the upstream activating sequence (UAS), directing the EBP to a specific σ^54^ promoter. The ATPase domain of the EBP then catalyzes ATP hydrolysis, leading to open complex formation and transcription. Without this co-activation step, transcription cannot occur. High levels of EBPs have been shown *in vivo* and *in vitro* to enable formation of the active open complex independent of EBP binding to the UAS [51], [52].

As our data supports direct σ^54^-mediated transcription of the operon containing *oxyB* in the heterologous host *E. coli*, co-activation must be occurring. σ^54^ over-expression has been shown to positively regulate many of the twelve EBPs native to *E. coli* [53], including *glnG*, *zraR*, and *fhlA* [30]. As no EBPs are present in the oxytetracycline gene cluster (no open reading frames in the oxytetracycline gene cluster possess the key AAA+ and helix-turn-helix domains found in EBPs [53]), a potential hypothesis for co-activation in our heterologous transcription is the UAS-independent co-activation by a positively regulated native *E. coli* EBP.

Over-expression of σ^S^ and FecI showed an increase in *oxyB* transcription but did not lead to detectable levels of oxytetracycline production. *oxyB* transcript levels were at least 10 fold lower for σ^S^ and FecI over-expression than when compared to σ^54^ over-expression. Presumably the low levels of the *oxyB* transcript were insufficient to generate enough OxyB to produce detectable levels of oxytetracycline. A plausible explanation for the transcription of *oxyB* during σ^S^ and FecI over-expression is cross-talk between these alternative sigma factors and σ^54^, leading to low levels of σ^54^-mediated transcription. This is supported by the observation that σ^54^, σ^S^, and FecI are known to be linked by the polyamine response [54], [55].

The identification of a functional σ^54^ promoter in the *S. rimosus* oxytetracycline biosynthetic gene cluster and 236 putative σ^54^ promoters in 61 gene clusters from seven different actinobacterial genomes is highly unexpected (Table S3). *Actinobacteria* do not contain a gene encoding σ^54^. Three hypotheses could account for this observation. These promoters are non-functional in *Actinobacteria* and are remnants of horizontal biosynthetic pathway transfer from non-*Actinobacteria* where these σ^54^ promoters are functional. Alternatively, a functional equivalent of σ^54^ could be present. Extensive research efforts into regulation of polyketide biosynthesis in *Streptomyces* has not uncovered a functional equivalent to σ^54^, however the vast majority of these studies have been carried out in *S. coelicolor*, the only *Streptomyces* in our genome analysis to contain no σ^54^ promoters in any of its 10 polyketide and non-ribosomal peptide biosynthetic gene clusters (Table S3). A third hypothesis is that these promoters represent false positives from the bioinformatics-based σ^54^ promoter prediction. While the prediction of promoters for orthologs across a wide range of bacterial species, as is done in this study, can decrease the false positive rate associate with PWM-based analyses and improve the selectivity of genome wide predictions, experimentation is ultimately required to evaluate the functionality of individual promoters. Understanding the origins and roles of these putative σ^54^ promoter sequences thus remains a wide-open question.

We have developed a new *E. coli*-based heterologous expression system for oxytetracycline polyketide biosynthetic gene clusters. We have demonstrated the over-expression of the alternative sigma factor σ^54^ directly and positively regulates heterologous expression of the oxytetracycline biosynthetic gene cluster in *E. coli*. Bioinformatics analysis indicates that σ^54^ promoters are present in nearly 70% of polyketide and non-ribosomal peptide biosynthetic pathways suggesting that σ^54^-mediated heterologous expression may be an effective, general approach. If sufficiently general, this approach may facilitate heterologous expression of new polyketides from metagenomic and environmental DNA samples.

This study also opens the door to characterizing and engineering polyketide biosynthetic pathways in *E. coli.* The vast majority of genetic experiments, such as deletion or complementation experiments, used to characterize these biosynthetic pathways [22], [56]–[61] have been carried out in either native producing organisms or in *Streptomyces* heterologous hosts. By developing an *E. coli*-based heterologous expression system, these experiments can be carried out in the highly genetically malleable *E. coli*, simplifying these studies.

This study represents the first example of successfully expressing functional type II polyketide synthases in *E. coli* [22], [33]. Type II polyketide synthases have been extremely challenging to produce in *E. coli*, even when placed under the control of known *E. coli* promoters. A major hurdle to production of type II PKS in E. coli has been the ability to generate soluble KS-CLF dimer, an essential part of the polyketide biosynthetic machinery. When expressed as standalone or fusion proteins, KS and CLF have always formed insoluble inclusion bodies [22], [33]. The prevailing hypothesis for this result is an incompatibility between the rates of protein synthesis, subunit folding, and heterodimerization [33]. Our work shows that while it is possible to generate soluble functional KS and CLF from the oxytetracycline pathway, the KS-CLF-heterodimer is expressed at very low levels, limiting this tools utility as a recombinant protein expression system. Our results will however be of use for in vivo characterization of aromatic polyketide backbone construction and the ensuing tailoring chemistries.

Finally, it is not unreasonable to suggest that over-expression of other sigma factors, such as σ^H^, σ^S^ or FecI, may positively regulate transcription of other biosynthetic gene clusters, potentially expanding the scope of alternative sigma factor-mediated heterologous expression.

## Materials and Methods

### General

*Escherichia coli* XL1 Blue (Stratagene) and *E. coli* TOP10 (Invitrogen) cells were used for routine cloning and plasmid preparations. *E. coli* BAP1 was used in all heterologous production experiments. *E. coli* cells were grown in LB media (Fisher) supplemented with the appropriate antibiotics when necessary. Oxytetracyline standard and IPTG were purchased from Sigma-Aldrich. Antibiotics and media components were purchased from Fisher. All strains were chemically or electronically transformed using standard transformation protocols.

### Plasmid Construction

PrimeSTAR HS polymerase and the Thermocycler Mastercycler personal were used for all PCR reactions. Pfu Ultra II polymerase and the Thermocycler Mastercycler personal were used for mutagenesis reactions. Primers can be found in the expanded experimental procedures in the supplementary information (File S1). *E. coli* MG1655 genomic DNA was used as the template. Each sigma factor was cloned into pCR-Blunt and confirmed by sequencing. Genes were sub-cloned using NdeI and EcoRI restriction sites into pET28b or pKH22 (a pET21c derivative with an AvrII site engineered after the native EcoRI site) [62], except for *rpoE* and *rpoD*, where HindIII instead of EcoRI and NheI instead of NdeI were used to due presence of native restriction sites. To construct pDCS61 the *oxyTA1ABCD* cassette was amplified from pMRH08 [12] using forward primer 5′ – TAATACGACTCACTATAGGG – 3′ and the reverse primer 5′ – CAGTGAATT**C**TCATAGCTCCAGGCTG - 3′. The *oxyTA1ABCD* cassette was then digested with EcoRI and inserted into pET28b. Site directed mutagenesis was then performed on pDCS61 using forward primer 5′ – CCTGCGTCCCCT**AA**AACGGCGGTGGC – 3′ and the reverse primer 5′ – GCCACCGCCGTT**TT**AGGGGACGCAGG – 3′ following the QuikChange mutagenesis protocol to construct pDCS62. The mutant was confirmed by sequencing. To construct pLRVP09, pBR322 was digested with ScaI and SspI to remove the β-lactamase marker and ligated closed. Plasmid names and descriptions can be found in the expanded experimental procedures in the supplementary information (File S1).

### qPCR Analysis

Strains examined were BAP1+pDCS11, BAP1+pMRH08, BAP1+pMRH08+either pDCS11, pDCS57, pDCS58, pDCS59, pNDP6, or pNDP7, and BAP1+pDCS11+either pDCS61 or pDCS62. All strains were grown in 25 mL LB in shake flasks at 37°C. At O.D._600_ = 0.4, cultures were induced with 1 mM IPTG and incubated at 20°C for 24 hours. Cells were harvested for RNA isolation. In concert with RNA isolation the broth from each culture was extracted and analysed for oxytetracycline production as described below. The SV Total RNA Isolation System was used to isolate RNA. cDNA was generated from RNA using AMV Reverse Transcriptase. qPCR experiments were performed in triplicate for each strain with a 10-fold dilution series of the 16 s RNA acting as an internal standard. Primers were designed to provide 180–210 bp amplicons of the *rpoN*, *oxyB*, *oxyF*, *oxyK*, *oxyP*, and *oxyT* transcripts. qPCR primer sequences can be found in the expanded experimental procedures in the supplemental information (File S1). The qPCR reactions contained 1.0 µM of each primer, 12.5 µL Absolute SYBR Green QPCR mix, 0.5 µL prepared cDNA, and 10.5 µL dH_2_O to give a total reaction volume of 20 µL. A Mx3000 qPCR Thermocycler was used with the following conditions: 1 cycle 95°C for 15 min, 40 cycles of 95°C for 15 s, 55°C for 30 s, and 72°C for 30 s, followed by denaturation for 1 cycle at 95°C for 1 min, 55°C for 30 s, and 95°C for 30 s. Standard curve R^2^ values and amplification efficiency values ranged from 0.991–1.0 and 90.7%→100% respectively. The amplification efficiency was calculated using the formula A = 10^(−1/slope)^ in which the slope was calculated by regression analysis obtained from the C_t_ values versus calculating log number of cells in serial dilutions (See Table S1).

### Oxytetracycline Production, Isolation, Characterization

The negative control strains were prepared by electroporating *E. coli* BAP1 with either pDCS11, pDCS57, pDCS58, pDCS59, pNDP6, or pNDP7. These strains lacked the oxytetracycline pathway and thus cannot produce oxytetracycline. Test strains were prepared by electroporation of *E. coli* BAP1 with pMRH08, the oxytetracycline gene cluster, and either no additional plasmid, pDCS11, pDCS57, pDCS58, pDCS59, pNDP6, or pNDP7. Tetracycline resistant strains were generated by transforming electrocompetent BAP1/pMRH08 with pDCS11 and pLRVP09. All strains were grown in 25 mL LB medium with appropriate antibiotics in shake flasks at 37°C. At O.D._600_ = 0.4, cultures were induced with 1 mM IPTG and grown at 20°C for 48 hours. Cultures were treated with acetone (1 mL) and vigorously vortexed. Cell debris was removed by centrifugation and Amberlite XAD-16 resin (0.50 g) was added to the clarified media. The resin was incubated with the media overnight and collected by filtration. The organic extracts were eluted from the resin with 20 mL MeOH over 2 hours and concentrated *in vacuo*. Extracts were then resuspended in 100 µL MeOH for ESI-LC-MS/MS analysis. All ESI-LC-MS/MS analysis was performed on an API2000 LC/MS/MS System equipped with a turbo-ion spray ESI probe interfaced with a Prominence UFLC. A reverse phase BDS Hypersil C18 column (100 mm×2.1 mm I.D., 3 µm particle size,) was employed. Mobile phases throughout experimental were A: 5% MeCN∶95% H_2_O with 0.05% formic acid and B: 95% MeCN∶5% H_2_O with 0.05% formic acid. For initial determination of oxytetracycline production a gradient program (3 min 100% A, 40 min linear gradient to 100% B, 10 min 100% B) was developed with a flow rate of 0.250 mL/min into the mass spectrometer (Ion spray 5,500 V, mass range 250–550 m/z). For oxytetracycline MS^2^ fragmentation scans a gradient program (3 min 100% A, 30 min linear gradient to 100% B, 2 min 100% B) was developed and the mass spectrometer settings were optimized for oxytetracycline (Ion spray 5,500 V, collision energy 50 eV, Q3 scan 400–500 m/z, MS^2^ parent ion 461.3 m/z, MS^2^ product ion range 198–462 m/z). For MRM quantification of oxytetracycline production, a gradient program identical to that of the MS^2^ program was use with the following mass spectrometer settings: Ion spray 5,500 V, collision energy 33 eV, MRM Q1 461.3 m/z, MRM Q3 483.2 m/z. Quantification was performed in triplicate using a standard curve generated from authentic oxytetracycline.

### Analysis of protein over-expression

BAP1, BAP1/pDCS11, and BAP1/pDCS11/pMRH08 were each grown in 25 mL LB medium with appropriate antibiotics in shake flasks at 37°C. At O.D._600_ = 0.4, cultures were induced with 1 mM IPTG and grown at 20°C for 24 hours. Cells were harvested from 5 mL of cell culture by centrifugation at 4000 g and resuspended in 200 µL of lysis buffer (100 mM sodium

Phosphate pH 8.0, 300 mM NaCl, 10% (*v*/*v*) glycerol, 1 mg/mL lysozyme, 1 µg/mL pepstatin A, and 1 µg/mL leupeptin). The cells were disrupted by sonication on ice, and cell debris was removed by centrifugation at 15000 g and 4°C. The resulting soluble fraction was analyzed by 4–20% gradient SDS-PAGE followed by staining with Coomassie Brilliant Blue.

### Gene cluster identification

To identify polyketide and non-ribosomal peptide biosynthetic gene clusters from bacterial genomes, all genes with cluster of orthologous groups (COG) annotations [35] of polyketide synthase (COG3321), 3-oxoacyl acyl carrier protein synthase (COG0304), and non-ribosomal peptide synthetase (COG1020) were identified from 58 bacterial genomes. A list of genomes used can be found in the expanded experimental procedures in the supplementary information (File S1). COG0304 annotated genes with gene names containing *fab* were discarded as they are involved in fatty acid biosynthesis. Operons containing genes commonly associated with secondary metabolite biosynthesis were included in the gene cluster. Transposons and housekeeping genes were considered to occur outside of the biosynthetic gene cluster. All identified gene clusters can be found in the supplementary information (Table S2).

### σ^54^ promoter identification

To identify putative σ^54^ promoter sequences in bacterial genomes the PromScan Perl script [32] was modified to output all hits, including intragenic hits, with a score of 65 or higher. The complete script can be found in the expanded experimental procedures in the supplementary information (File S1). The script was run on Windows using Strawberry Perl (http://strawberryperl.com/). The inputs were bacterial genome DNA sequence files (FNA files from NCBI's Genome Database) and the σ^54^ promoter positional weighted matrix file, which is based on 186 known σ^54^ promoter sites and can be found in the expanded experimental procedures in the supplementary information [33].

The results were uploaded to an SQL database created using SQL Server 2008 Express (http://www.microsoft.com/express/). PTT files (from NCBI's Genome Database) containing annotation data were uploaded to the database. Using LINQ to SQL, hits generated from the modified PromScan algorithm were linked to genes located within 500 bp upstream and in the same coding direction as the gene start site. Promoter prediction data is available on the web at http://www.sigma54.ca.

### 5′ RACE

Total RNA was isolated from BAP1 with pMRH08 and pDCS11 as described for qPCR analysis. cDNA was generated using AMV Reverse Transcriptase and the *oxyA* specific primer 5′ – AGGCTCATCGTCATGCCGCA – 3′, Poly(dC) tails were added using terminal deoxynucleotidyl transferase (Fermentas). The tailed cDNA was amplified by PCR by PrimeStar HS DNA polymerase (Takara) using the *oxyA* specific primer and the abridged anchor primer 5′ – GGCCACGCGTCGACTAGTACGGGIIGGGIIGGGII – 3′. The diluted PCR reaction was used as the template for a second round of PCR amplification using the *oxyA* nested primer 5′ – CCGCCTCGCCCGTCACAC – 3′ and the abridged universal anchor primer 5′– GGCCACGCGTCGACTAGTAC – 3′. The PCR products were gel purified and reamplified using the *oxyA* nested primer and the abridged universal anchor primers. The PCR products were then cloned into pCR-Blunt and sequenced.

## Supporting Information

Figure S1 **Transcript levels, determined by qPCR, for** ***E. coli*** **transformed with the oxytetracycline gene cluster (pMRH08) compared to a null strain lacking the oxytetracycline gene cluster.**
(TIF)

Figure S2 **Transcript levels, determined by qPCR, for** ***E. coli*** **transformed with the oxytetracycline gene cluster (pMRH08) and over-expressing σ^54^ compared to a null strain lacking the oxytetracycline gene cluster and over-expressing σ^54^.**
(TIF)

Figure S3 **ESI-LC-MS/MS Q1 ion extraction chromatograms for the oxytetracycline [M+H]^+^** ***m*** **/** ***z*** **= 461.** **Standard**: an oxytetracycline standard. **Positive control**: Organic extracts from a culture of BAP1 with the oxytetracycline biosynthetic gene cluster over-expressing σ^54^. **Negative control**: organic extracts from a culture of BAP1 with the oxytetracycline biosynthetic gene cluster. **σ^E^**: organic extracts from a culture of BAP1 with the oxytetracycline biosynthetic gene cluster over-expressing σ^E^. **σ^F^**: organic extracts from a culture of BAP1 with the oxytetracycline biosynthetic gene cluster over-expressing σ^F^. **σ^H^**: organic extracts from a culture of BAP1 with the oxytetracycline biosynthetic gene cluster over-expressing σ^H^. MS^2^ traces further confirm that no oxytetracycline can be detected in the σ^E^, σ^F^, and σ^H^ samples.
(TIF)

Figure S4 **SDS-PAGE of the soluble fraction from oxytetracycline producing strains.** Lane 1 is protein ladder, lane 2 is the soluble fraction from uninduced BAP1, lane 3 is the solubule fraction 24 h post induction from BAP1/pDCS11, lane 4 is the soluble fraction 24 h post induction from BAP1/pMRH08/pDCS11. A band corresponding to σ^54^ (54 kDa) is seen in lanes 3 and 4. The OxyA-OxyB KS-CLF heterodimer (45 kDa and 44 kDa respectively) is not obeserved in lane 4.
(TIF)

File S1 **Expanded experimental procedures.**
(PDF)

Table S1 **Complete data set for qPCR analysis as shown in** **Figures 1** **and** **3** **.** Standard curve, efficiencies, R^2^ and Ct values are given.
(PDF)

Table S2 **List of putative σ^54^ promoters identified from a bioinformatics analysis of the oxytetracycline gene cluster.**
(TIF)

Table S3 **List of PKS, NRPS and NRPS/PKS gene clusters with the associated σ^54^ promoters identified from our bioinformatics analysis.**
(PDF)

Table S4 **Tabulated number of PKS, NRPS and NRPS/PKS gene clusters with one of more σ^54^ promoters identified from our bioinformatics analysis at increasing cutoff stringencies.**
(TIF)
