# Supplementary figures and images for: Alternative Sigma Factor Over-Expression Enables Heterologous Expression of a Type II Polyketide Biosynthetic Pathway in Escherichia coli
Source: PLoS One. 2013 May 28;8(5):e64858. doi: 10.1371/journal.pone.0064858 (PMC3665592; doi:10.1371/journal.pone.0064858)

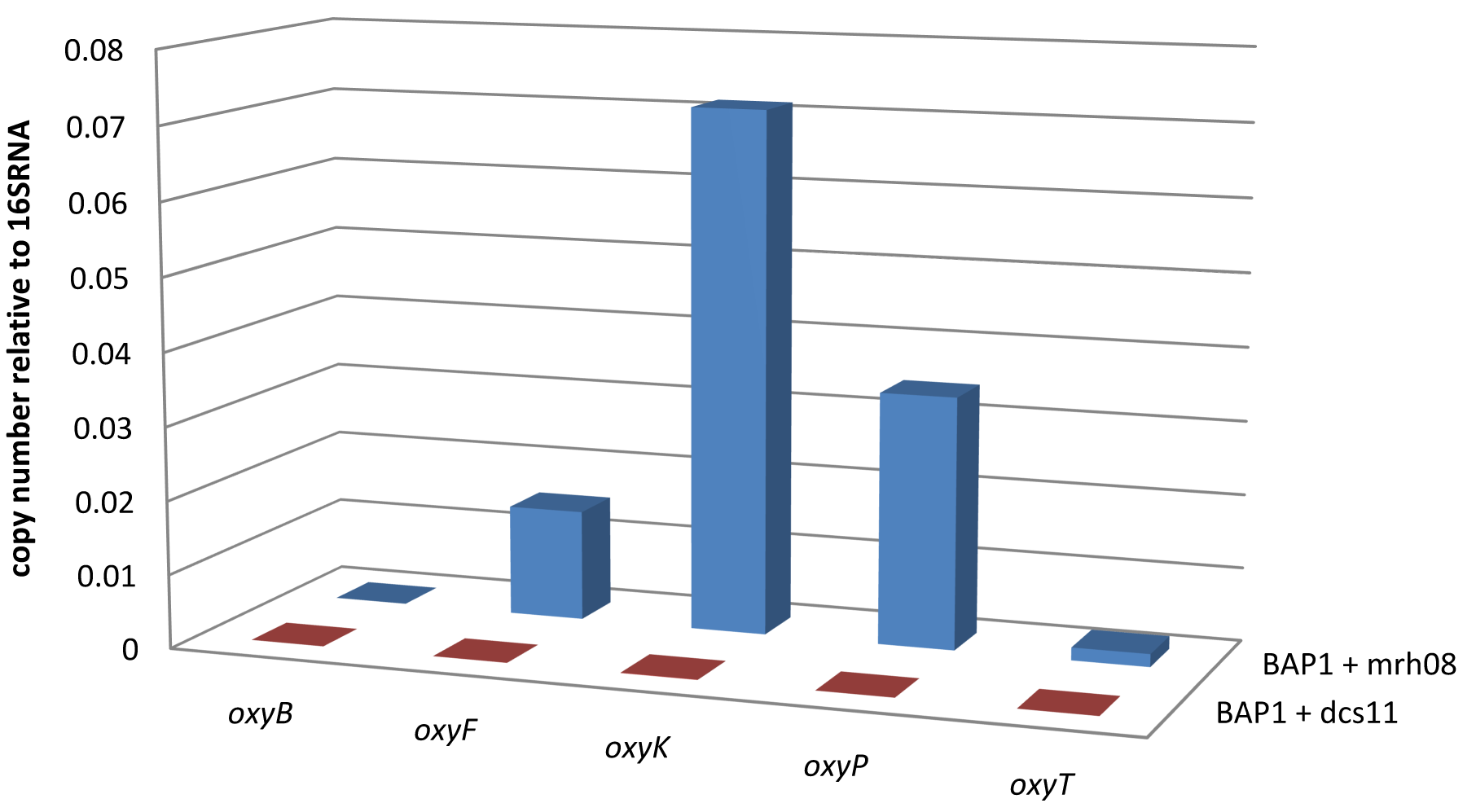

Supplement: Figure S1 — Transcript levels, determined by qPCR, for E. coli transformed with the oxytetracycline gene cluster (pMRH08) compared to a null strain lacking the oxytetracycline gene cluster. (TIF) [file pone.0064858.s001.tif]

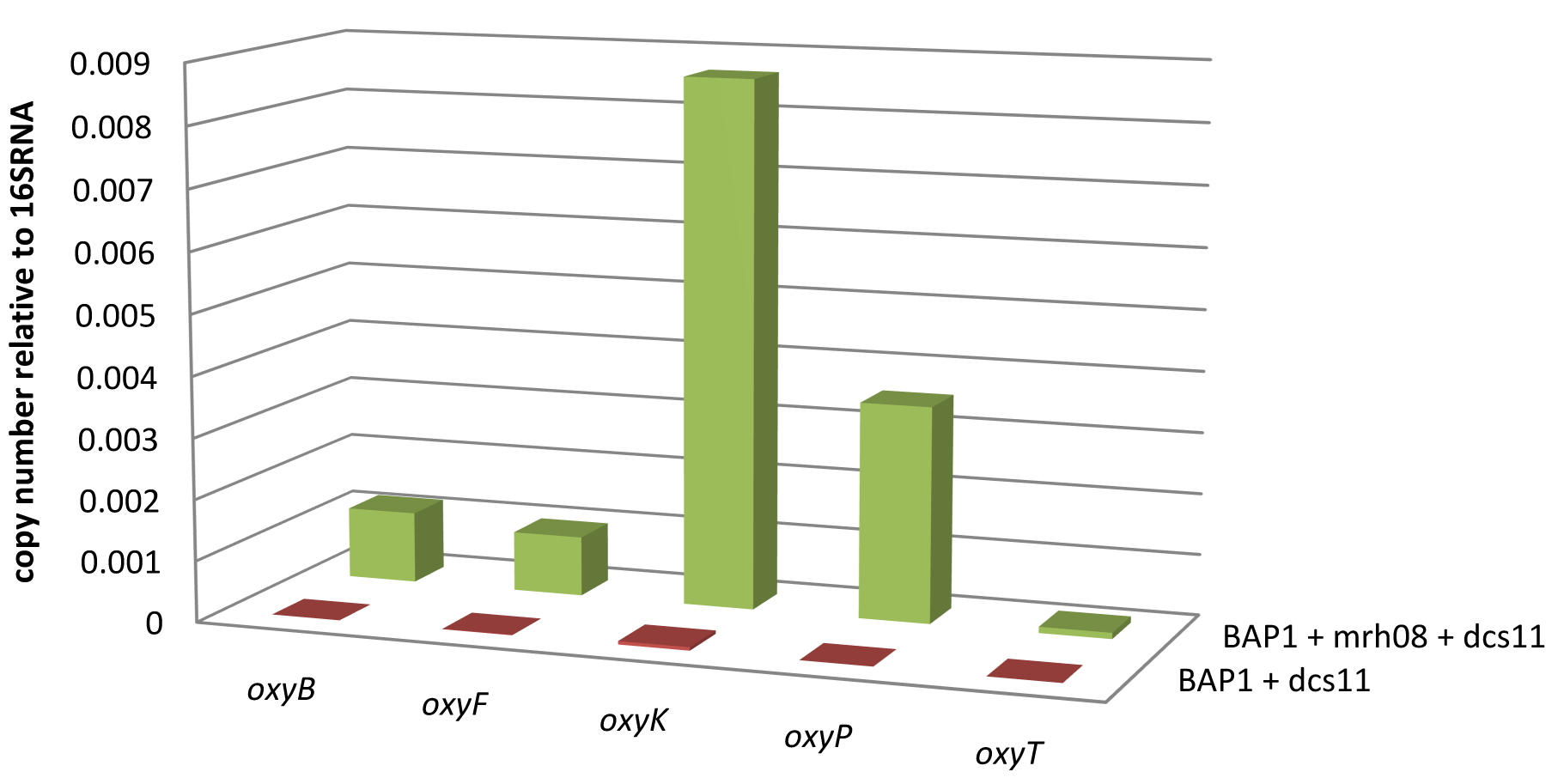

Supplement: Figure S2 — Transcript levels, determined by qPCR, for E. coli transformed with the oxytetracycline gene cluster (pMRH08) and over-expressing σ54 compared to a null strain lacking the oxytetracycline gene cluster and over-expressing σ54. (TIF) [file pone.0064858.s002.tif]

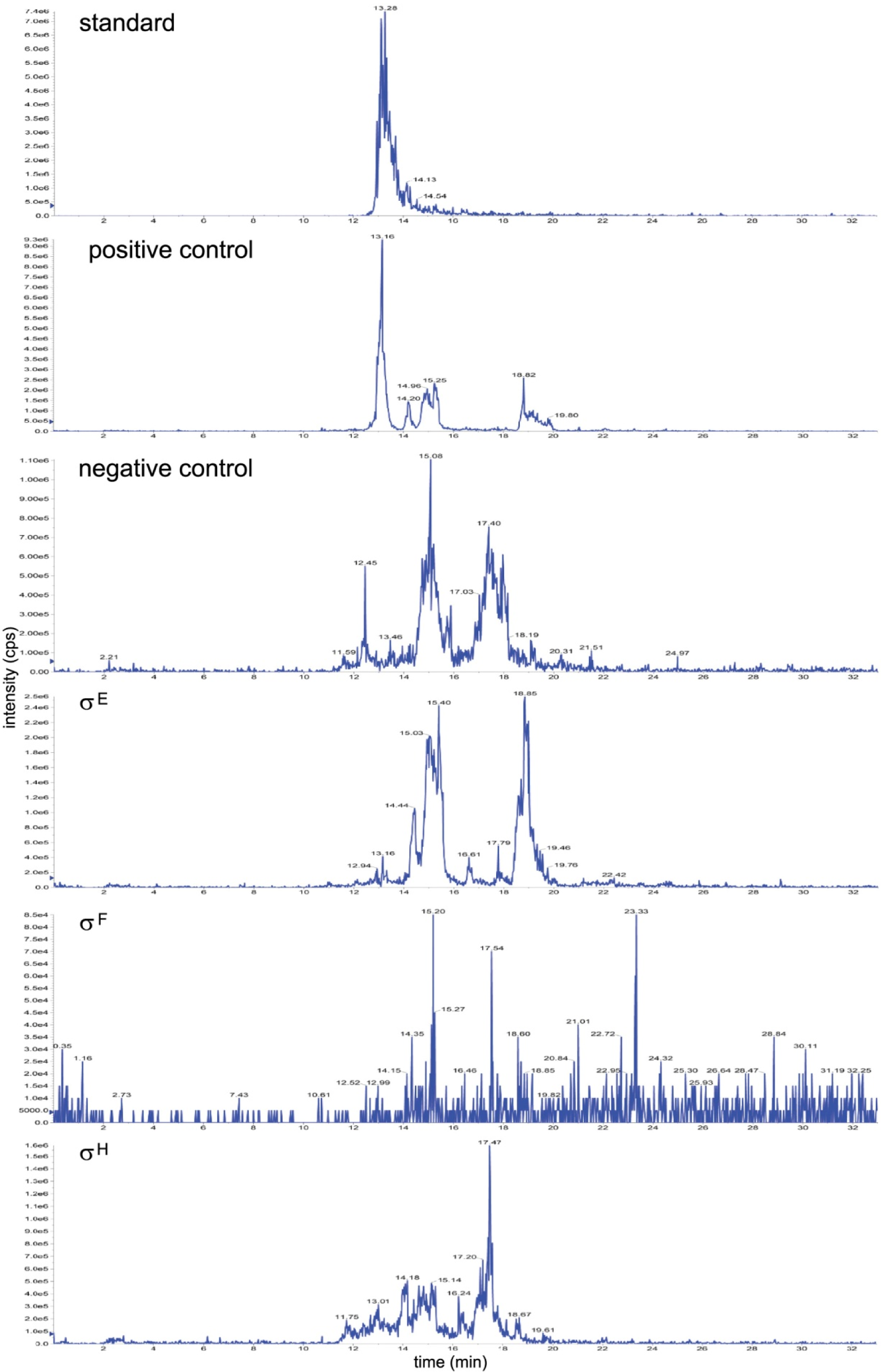

Supplement: Figure S3 — ESI-LC-MS/MS Q1 ion extraction chromatograms for the oxytetracycline [M+H]+ m / z = 461. Standard: an oxytetracycline standard. Positive control: Organic extracts from a culture of BAP1 with the oxytetracycline biosynthetic gene cluster over-expressing σ54. Negative control: organic extracts from a culture of BAP1 with the oxytetracycline biosynthetic gene cluster. σE: organic extracts from a culture of BAP1 with the oxytetracycline biosynthetic gene cluster over-expressing σE. σF: organic extracts from a culture of BAP1 with the oxytetracycline biosynthetic gene cluster over-expressing σF. σH: organic extracts from a culture of BAP1 with the oxytetracycline biosynthetic gene cluster over-expressing σH. MS2 traces further confirm that no oxytetracycline can be detected in the σE, σF, and σH samples. (TIF) [file pone.0064858.s003.tif]

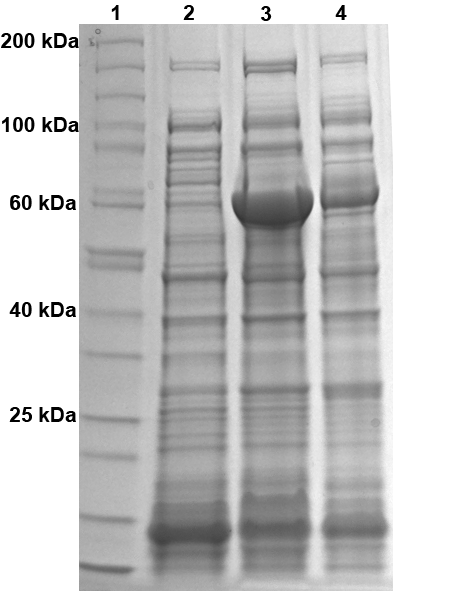

Supplement: Figure S4 — SDS-PAGE of the soluble fraction from oxytetracycline producing strains. Lane 1 is protein ladder, lane 2 is the soluble fraction from uninduced BAP1, lane 3 is the solubule fraction 24 h post induction from BAP1/pDCS11, lane 4 is the soluble fraction 24 h post induction from BAP1/pMRH08/pDCS11. A band corresponding to σ54 (54 kDa) is seen in lanes 3 and 4. The OxyA-OxyB KS-CLF heterodimer (45 kDa and 44 kDa respectively) is not obeserved in lane 4. (TIF) [file pone.0064858.s004.tif]

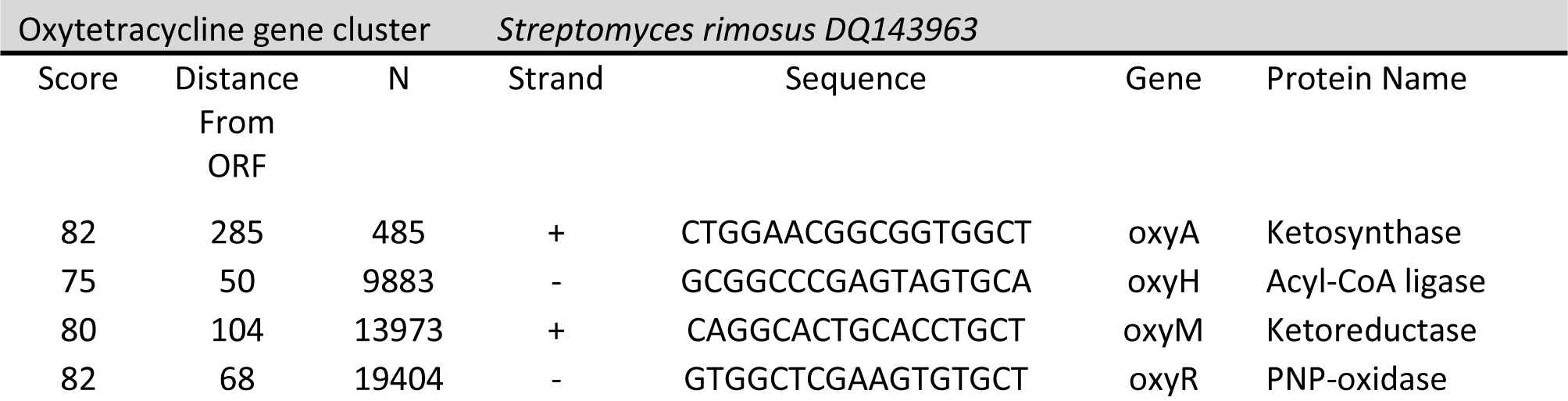

Supplement: Table S2 — List of putative σ54 promoters identified from a bioinformatics analysis of the oxytetracycline gene cluster. (TIF) [file pone.0064858.s007.tif]

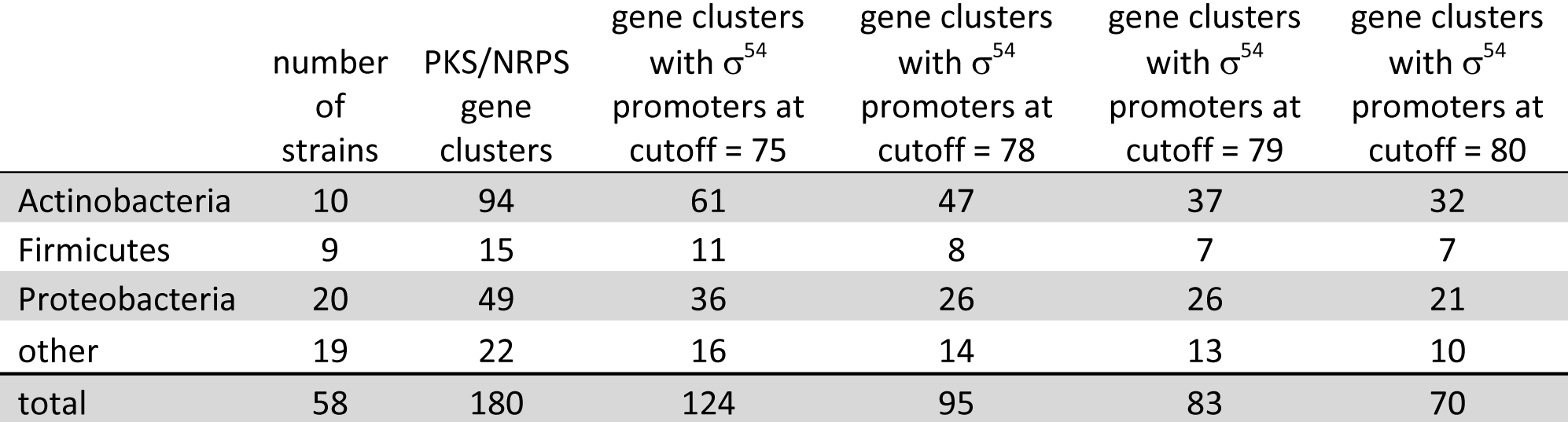

Supplement: Table S4 — Tabulated number of PKS, NRPS and NRPS/PKS gene clusters with one of more σ54 promoters identified from our bioinformatics analysis at increasing cutoff stringencies. (TIF) [file pone.0064858.s009.tif]
