# Supplementary material for: Alternative Sigma Factor Over-Expression Enables Heterologous Expression of a Type II Polyketide Biosynthetic Pathway in Escherichia coli
Source: PLoS One. 2013 May 28;8(5):e64858. doi: 10.1371/journal.pone.0064858 (PMC3665592; doi:10.1371/journal.pone.0064858)
Supplement: File S1 — Expanded experimental procedures. (PDF) [file pone.0064858.s005.pdf]

Alternative Sigma Factor Over-Expression Enables Heterologous Expression of a Type II Polyketide Biosynthetic Pathway in *Escherichia coli*

David Cole Stevens, Kyle R. Conway, Nelson Pearce, Luis Roberto Villegas-Peñaranda, Anthony Garza and Christopher N. Boddy*

File S1. Epanded experimental procedures.

Cloning, PCR amplification, and site directed mutagenesis.

A complete list of the plasmids used in this study.

PLASMID	DESCRIPTION
DCS08	*E. coli rpoN* in pCR-BLUNT
DCS11	*E. coli rpoN* in pKH22
DCS53	*E. coli rpoF* in pCR-BLUNT
DCS54	*E. coli rpoE* in pCR-BLUNT
DCS55	*E. coli fecI* in pCR-BLUNT
DCS57	*E. coli rpoF* in pKH22
DCS58	*E. coli rpoE* in pKH22
DCS59	*E. coli fecI* in pKH22
DCS61	*S. rimosus oxyABCD* cassette in pET28b
DCS62	DCS61 with *oxyAp* Δ-24 mutation
NDP4	*E. coli rpoS* in pCR-BLUNT
NDP5	*E. coli rpoH* in pCR-BLUNT
NDP6	*E. coli rpoS* in pKH22
NDP7	*E. coli rpoH* in pKH22
MRH08	*S. rimosus* oxytetraycline biosynthetic pathway in pET28b
LRVP09	pBR322 lacking *BLA*

A complete list of the primers used for PCR amplification and mutagenesis.

GENE	FORWARD	REVERSE	PROTEIN
E. coli rpoN	aatta**catatg**aacgaacctttgcaactca	aatta**gaattc**tcaaacgagtgtttacg	σ^{54}
E. coli rpoD	aatt**gctagc**atggagcaaaacccgc	aatt**gaattc**ttaattcgtccaggaag	σ^{70}
E. coli rpoE	aatt**catatg**agcgagcagttaacgg	aatt**gaattc**tcaacgcctgataagc	σ^{E}
E. coli rpoF	aatt**catatg**gtgaattcactctatac	aatt**aagctt**ttataacttacccagtt	σ^{F}
E. coli fecI	aatt**catatg**tctgaccgcgccacta	aatt**gaattc**tcaacgcctgataagc	FecI
E. coli rpoH	gtga**catatg**actgacaaaatgcaaa	cacg**gaattc**ttacgcttcaatggca	σ^{H}
E. coli rpoS	aatta**catatg**aagcaaggtttgcaactca	aatta**gaattc**tcaaacgagttgtttacg	σ^{S}
S. rimosus oxyABCD	T7 promoter primer	cagt**gaattc**tcatagctccaggctg	oxyA,B,C,D
oxyAp Δ-24 mutation	cctgcgtcccct**aa**aacggcggtggc	gccaccgccgtt**tt**aggggacgcagg	N/A

qPCR study.

A complete list of the primers used for qPCR analysis.

TRANSCRIPT	FORWARD	REVERSE
16s RNA	aactgcctgatggagggggg	gtctcagttccagtgtggct
rpoN	gtctgttgcagttgtcgacg	cggtgtaaatggtgtcccaa
oxyB	tcaccggcctcggtgtggtc	gcgcgagccgggtcatcagg
oxyF	cccgcctgctccaccagcgc	cctggagtgggacgacgaga
oxyK	ccctgatcagggacgccgcc	tcgcggccaccggaggcgaa

oxyP	ggcccatccgcccgcttagg	gcaggtcgccgaccccgcgt
oxyT	aggcgaaggccctgcacagc	cggacaccaggaacgcctgg

5' RACE.

A complete list of primers used for the 5' RACE experiment

Primer name	sequence
oxyA_RACE2	aggctcatcgtcatgccgca
oxyA_nested	ccgcctcgcccgtcacac
AAP	ggccacgcgtcgactagtacgggiigggiigggii
AUAP	ggccacgcgtcgactagtac

Bioinformatics

A list of organisms used in the bioinformatics analysis.

Phylum	Genome Name	Contains σ^{54}	RefSeq ID
Actinobacteria	Bifidobacterium longum NCC2705, complete genome	N	NC_004307.2
Actinobacteria	Mycobacterium tuberculosis CDC1551, complete genome	N	NC_002755.2
Actinobacteria	Mycobacterium ulcerans Agy99, complete genome	N	NC_008611.1
Actinobacteria	Nocardia farcinica IFM 10152, complete genome	N	NC_006361.1
Actinobacteria	Rubrobacter xylanophilus DSM 9941, complete genome	N	NC_008148.1
Actinobacteria	Saccharopolyspora erythraea NRRL 2338, complete genome	N	NC_009142.1
Actinobacteria	Salinispora tropica CNB-440, complete genome	N	NC_009380.1
Actinobacteria	Streptomyces avermitilis MA-4680, complete genome	N	NC_003155.4
Actinobacteria	Streptomyces coelicolor A3(2), complete genome	N	NC_003888.3
Actinobacteria	Streptomyces griseus subsp. griseus NBRC 13350, complete genome	N	NC_010572.1
Aquificae	Sulfurihydrogenibium sp. YO3AOP1, complete genome	Y	NC_010730.1
Bacteroidetes/Chlorobi	Bacteroides fragilis NCTC 9343, complete genome	Y	NC_003228.3
Bacteroidetes/Chlorobi	Candidatus Amoebophilus asiaticus 5a2, complete genome	Y	NC_010830.1
Bacteroidetes/Chlorobi	Gramella forsetii KT0803, complete genome	Y	NC_008571.1
Chlamydiae/Verrucomicrobia	Chlamydia trachomatis D/UW-3/CX, complete genome	Y	NC_000117.1
Chlamydiae/Verrucomicrobia	Chlamydophila pneumoniae AR39, complete genome	Y	NC_002179.2
Chlamydiae/Verrucomicrobia	Coraliomargarita akajimensis DSM 45221 chromosome, complete genome	N	NC_014008.1
Chloroflexi	Sphaerobacter thermophilus DSM 20745 chromosome 1, complete genome<br>Sphaerobacter thermophilus DSM 20745 chromosome 2, complete genome	Y	NC_013523.1<br>NC_013524.1
Crenarchaeota	Pyrobaculum arsenaticum DSM 13514, complete genome	N	NC_009376.1
Cyanobacteria	Gloeobacter violaceus PCC 7421, complete genome	N	NC_005125.1
Cyanobacteria	Microcystis aeruginosa NIES-843, complete genome	N	NC_010296.1
Cyanobacteria	Nostoc punctiforme PCC 73102, complete genome	N	NC_010628.1
Cyanobacteria	Synechocystis sp. PCC 6803, complete genome	N	NC_000911.1

Deinococcus-Thermus	Deinococcus deserti VCD115, complete genome	N	NC_012526.1
Euryarchaeota	Pyrococcus horikoshii OT3, complete genome	N	NC_000961.1
Fibrobacteres/Acidobacteria	Solibacter usitatus Ellin6076, complete genome	Y	NC_008536.1
Firmicutes	Bacillus cereus subsp. cytotoxis NVH 391-98, complete genome	Y	NC_009674.1
Firmicutes	Bacillus subtilis subsp. subtilis str. 168, complete genome	Y	NC_000964.3
Firmicutes	Clostridium acetobutylicum ATCC 824, complete genome	Y	NC_003030.1
Firmicutes	Clostridium kluyveri DSM 555, complete genome	Y	NC_009706.1
Firmicutes	Clostridium perfringens str. 13, complete genome	Y	NC_003366.1
Firmicutes	Lactobacillus brevis ATCC 367, complete genome	N	NC_008497.1
Firmicutes	Listeria monocytogenes str. 4b F2365, complete genome	Y	NC_002973.6
Firmicutes	Staphylococcus aureus subsp. aureus MRSA252, complete genome	N	NC_002952.2
Firmicutes	Streptococcus pyogenes M1 GAS, complete genome	N	NC_002737.1
Proteobacteria (Alpha)	Agrobacterium tumefaciens str. C58 chromosome circular, complete sequence<br>Agrobacterium tumefaciens str. C58 chromosome linear, complete sequence	Y	NC_003062.2<br>NC_003063.2
Proteobacteria (Alpha)	Methylobacterium extorquens DM4, complete genome	Y	NC_012988.1
Proteobacteria (Alpha)	Rickettsia rickettsii str. Iowa, complete genome	N	NC_010263.1
Proteobacteria (Beta)	Neisseria meningitidis MC58, complete genome	Y	NC_003112.2
Proteobacteria (Beta)	Ralstonia solanacearum GMI1000 plasmid pGMI1000MP, complete sequence<br>Ralstonia solanacearum GMI1000, complete genome	Y	NC_003296.1<br>NC_003295.1
Proteobacteria (Delta)	Geobacter sulfurreducens PCA, complete genome	Y	NC_002939.4
Proteobacteria (Delta)	Myxococcus xanthus DK 1622, complete genome	Y	NC_008095.1
Proteobacteria (Delta)	Sorangium cellulosum 'So ce 56', complete genome	Y	NC_010162.1
Proteobacteria (Epsilon)	Campylobacter jejuni subsp. jejuni NCTC 11168, complete genome	Y	NC_002163.1
Proteobacteria (Epsilon)	Helicobacter pylori J99, complete genome	Y	NC_000921.1
Proteobacteria (Gamma)	Candidatus Carsonella ruddii PV, complete genome	N	NC_008512.1
Proteobacteria (Gamma)	Escherichia coli str. K-12 substr. MG1655, complete genome	Y	NC_000913.2
Proteobacteria (Gamma)	Francisella tularensis subsp. tularensis SCHU S4, complete genome	N	NC_006570.2
Proteobacteria (Gamma)	Legionella pneumophila subsp. pneumophila str. Philadelphia 1, complete genome	Y	NC_002942.5
Proteobacteria (Gamma)	Pseudomonas aeruginosa PAO1, complete genome	Y	NC_002516.2
Proteobacteria (Gamma)	Pseudomonas putida KT2440, complete genome	Y	NC_002947.3
Proteobacteria (Gamma)	Pseudomonas syringae pv. tomato str. DC3000, complete genome	Y	NC_004578.1
Proteobacteria (Gamma)	Salmonella enterica subsp. enterica serovar Typhi str. CT18, complete genome	Y	NC_003198.1
Proteobacteria (Gamma)	Shewanella oneidensis MR-1, complete genome	Y	NC_004347.1
Proteobacteria (Gamma)	Yersinia pestis CO92, complete genome	Y	NC_003143.1
Spirochaetes	Borrelia burgdorferi B31, complete genome	Y	NC_001318.1
Spirochaetes	Treponema denticola ATCC 35405, complete genome	Y	NC_002967.9
Tenericutes	Candidatus Phytoplasma mali, complete genome	N	NC_011047.1

Positional weighted matrix used to describe the σ^{54} consensus sequence

A:	12	2	0	12	139	11	55	51	46	44	38	13	4	1	9	76
C:	14	0	0	147	23	122	17	48	64	42	62	22	18	2	173	5
G:	10	184	186	6	18	10	103	69	36	35	43	15	10	181	1	17
T:	150	0	0	21	6	43	11	18	40	65	43	136	154	2	3	88

Perl Script used to analyze genomes for putative σ^{54} promoters. The script below is a modified versiuon o fthe the original ProimScan perl script by David J. Studholme. It has been modified to facilitate promoter prediction in genomes of >5 Mb.

```
#!/usr/bin/perl -w

##################################################################################
#                                                                                #
#   copyright           : (C) 2000-2003 by David J. Studholme                    #
#   email               : ds2@sanger.ac.uk                                       #
#                                                                                #
##################################################################################

##################################################################################
#*                                                                               #
#*   This program is free software; you can redistribute it and/or modify      #
#*   it under the terms of the GNU General Public License as published by       #
#*   the Free Software Foundation; either version 2 of the License, or          #
#*   (at your option) any later version.                                        #
#*                                                                               #
##################################################################################

### VERSION / UPDATE LOG ###

# 7/11/02 Corrected bugs caught by Richard Pau
# 21/11/02 Changed to log base 2 rather than natural log
# 07/03 Enabled reporting of distance upstream
# enabled normalising of scores.
# 27/08/03 Switched format of matrix files.
# 27/08/03 Integrated Hit object into same file.

use strict;
use Getopt::Std;
use IO::File;
use IO::Handle;


$| = 1;

### Parse command line arguments
### -m specify matrix file, -g specify genome name, -c specify cut-off
### -h: help, -:statistics, -n: ptt, -i unknown , -m: matrix, -g: genome -c:cutoff
my %option;
getopts( "hsnim:g:c:", \%option );

if ( $option{h} ) {
        show_help();
        exit;
}

my ($seqread);
my ( $seq_length, @seq_window );
my ( $mean_score, $total_dev, $sd, $variance );
my $GC_content;

```

```
### Set threshold score above which counts as a hit
my $cut_off = 80;
if ( $option{c} ) {
	$cut_off = $option{c};
}

### Set input/output filenames
my $genome = "ecoli";
if ( $option{g} ) {
	$genome = $option{g};
}

### Remove any filename extensions
if ( $genome =~ m/([\w\d\.]+)\.fna/ ) {
	$genome = $1;
}

### Name of file containing sequence to be scanned
my $sequence_file = "$genome.fna";

### Name of file containing details of ORFs
my @ptt_file;
unless ( $option{n} ) {
	my $ptt_file = "$genome.ptt";
	my $ptt_fh   = new IO::File;
	$ptt_fh->open("<$ptt_file")
	  or die "Failed to open ptt file $genome.ptt: $!\n";
	while ( my $readline = <$ptt_fh> ) {
		push @ptt_file, $readline;
	}
	$ptt_fh->close;
}

### Name of matrix file
my $matrix_file = $option{m} or die "You must specify a matrix file\n";

### Open error file

open OUTPUT, '>', $option{g}.".txt" or die $!;
STDOUT->fdopen( \*OUTPUT, 'w' ) or die $!;


### Begin the scans!!
promscan_kl( $matrix_file, $sequence_file, \@ptt_file );
exit;

sub promscan_kl {

	my ( $matrix_file, $sequence_file, $ptt_file ) = @_;

	my %all_scores_forward;
	my %all_scores_reverse;
	my @hits;

	my ( $sequence, $header );
	print STDERR "Reading the sequence file into memory ... ";
```

```
my $seq_fh = new IO::File;
$seq_fh->open("<$sequence_file")
  or die "Failed to open $sequence_file: $!\n";

while ( my $readline = <$seq_fh> ) {
        chomp $readline;
        $sequence .= $readline if $readline =~ m/^[A-Za-z]+$/;
        if ( $readline =~ m/>(.*)/ ) {
                $header = $1;
        }
}
$seq_fh->close;

print STDERR "done.\n";
print STDERR "Sequence: $header\n\n";
print "Sequence|$header\n";

print STDERR "Splitting the sequence into a list ... ";
my @sequence = split //, $sequence;
print STDERR "done.\n";

### Calculate the GC content of the sequence
my $GC_content = calculate_GC(@sequence);
print "G+C|$GC_content\n";
print STDERR "G+C = $GC_content\n";
my %content;
$content{G} = $GC_content / 2;
$content{C} = $GC_content / 2;
$content{A} = ( 1 - $GC_content ) / 2;
$content{T} = ( 1 - $GC_content ) / 2;

### Read matrix file
my $pmatrix = read_matrix_file($matrix_file);
print "Matrix|".$option{m}."\n";
my %matrix  = %$pmatrix;


### Do sanity check on matrix, and calculate width of the matrix
die "Matrix corrupted\n"
  unless ( @{ $matrix{A} } == @{ $matrix{C} }
        and @{ $matrix{C} } == @{ $matrix{G} }
        and @{ $matrix{A} } == @{ $matrix{T} } );
my $seq_window_length = @{ $matrix{A} };

### Calculate maximum possible score
my $overall_highest = 0;

for ( my $i = 1 ; $i < $seq_window_length ; $i++ ) {
        my $highest_score = 0;
        my $highest_base;
        foreach my $base ( "A", "C", "G", "T" ) {
                if ( $matrix{$base}[$i] > $highest_score ) {
                        $highest_score = $matrix{$base}[$i];
                        $highest_base  = $base;
                }
        }
```

```
                $overall_highest +=
                  ( $highest_score * log( $highest_score / $content{$highest_base} ) /
                          log(2) )
                  if $highest_score;
        }

#print STDERR "(Highest possible score before normalisation = $overall_highest)\n" ;
#print "(Highest possible score before normalisation = $overall_highest)\n" ;

        print STDERR "Sites scoring > $cut_off\n";
###     print "Sites scoring > $cut_off\n";

        ### Initialise the first window of sequence to be scanned
        for ( my $i = 0 ; $i < $seq_window_length ; $i++ ) {
                $seq_window[$i] = $sequence[$i];
        }

        ### read sequence one base at a time
        my $n = 0;

        while ( my $seqread = uc( shift @sequence ) ) {
                my $seq_window;
                if ( $seqread =~ m/[ACGTN]/ ) {    # sanity-check for legal bases

                        ### move the sequence window along one residue
                        shift(@seq_window);           # removes first residue
                        push( @seq_window, $seqread ); # adds next residue

                        ### Assign a score to current window
                        ### This score, known as the Kullback-Liebler distance,
                        ### reflects the theoretical binding energy of the
                        ### DNA-protein interaction.
                        ### For more information see: Stormo, G.D. 2000.
                        ### DNA binding sites: representation and discovery.
                        ### Bioinformatics. 16:16-23.

                        ### First check the forward strand
                        my $strand = "+";

                        ### Calculate score. Remember, divide by log2 to get log to the base of 2
                        my $current_score = 0;
                        for ( my $i = 0 ; $i < $seq_window_length ; $i++ ) {
                                my $base = $seq_window[$i];
                                $current_score = $current_score + ( ( $matrix{$base}[$i] ) *
                                         log( $matrix{$base}[$i] / $content{$base} ) / log(2) )
                                  if $matrix{$base}[$i];
                        }

                        ### Normalise the score
                        $current_score /= $overall_highest;
                        $current_score *= 100;

                        if ( $option{s} ) {
                                ### Record each score in a matrix %all_scores_forward
                                $all_scores_forward{$n} = $current_score;
                        }
```

```
	### If this window scores greater than the cut off, then record details ....
	if ( $current_score > $cut_off ) {

		### Convert @seq_window into a string, $seq_window
		$seq_window = "";
		my $q = 0;
		while ( $seq_window[$q] ) {
			$seq_window .= $seq_window[$q];
			$q++;
		}

		### Is hit in coding or non-coding region?
		my ( $locate_match, $intergenic_size ) =
		  locate_match( $ptt_file, $n, $strand, $sequence,
			$seq_window );

		### Round score to nearest whole number
		$current_score = int($current_score);

		### Print details of this window
		if ( $locate_match or $option{n} ) {

			### Create a new Hit object and set its values
			my $hit = Hit->new();
			$hit->position($n);
			$hit->strand($strand);
			$hit->score($current_score);
			$hit->strand($strand);
			$hit->sequence($seq_window);
			$hit->locus($locate_match);

			### Record the existence of this Hit object in an array
			push @hits, $hit;

			printf STDERR "%8s|%1s|%s|%5d|%s\n", $n, $strand,
			  $seq_window, $current_score, $locate_match;
		}
	}

	### now check the Reverse strand
	$strand = "-";

	### Calculate score. Remember divide by log2 to get log to the base of 2
	$current_score = 0;
	for ( my $i = 0 ; $i < $seq_window_length ; $i++ ) {
		my $j    = $seq_window_length - $i - 1;
		my $base = revcomp( $seq_window[$j] );
		$current_score = $current_score + ( ( $matrix{$base}[$i] ) *
			  log( $matrix{$base}[$i] / $content{$base} ) / log(2) )
		  if $matrix{$base}[$i];
	}

	### Normalise the score
	$current_score /= $overall_highest;
	$current_score *= 100;
```

```
			if ( $option{s} ) {
				### Record each score in a matrix %all_scores_forward
				$all_scores_reverse{$n} = $current_score;
			}

			#### print current score if greater than threshold value
			if ( $current_score > $cut_off ) {

				### Convert @seq_window into a string, $seq_window
				$seq_window = "";
				my $q = 0;
				while ( $seq_window[$q] ) {
					$seq_window = $seq_window . $seq_window[$q];
					$q++;
				}

				### Convert $seq_window to Reverse Complement
				my $revcomp = reverse($seq_window);
				$revcomp =~ tr/ACGTacgt/TGCAtgca/;
				$seq_window = $revcomp;

				### Is hit in coding or non-coding region?
				my ( $locate_match, $intergenic_size ) =
				  locate_match( $ptt_file, $n, $strand, $sequence,
					$seq_window );

				### Round the score to nearest whole number
				$current_score = int($current_score);

				### Print details of this window
				### ... but only if it is intergenic
				if ( $locate_match or $option{n} ) {

					### Create a new Hit object and set its values
					my $hit = Hit->new();
					$hit->position($n);
					$hit->strand($strand);
					$hit->score($current_score);
					$hit->strand($strand);
					$hit->sequence($seq_window);
					$hit->locus($locate_match);

					### Record the existence of this Hit object in an array
					push @hits, $hit;

					printf STDERR "%8s|%1s|%s|%5d|%s\n", $n, $strand,
					  $seq_window, $current_score, $locate_match;
				}
			}

			### The sequence pointer proceeds by one base
			$n++;

		}
		;    # end of if($seqread =~ /[ACGTN]/)
```

```
	};	# end of while (read $sequence_file, $seqread, 1) loop

	### The final value of $n, the sequence-pointer,
	### gives the number of readable bases
	$seq_length = $n;
	### print "N = $seq_length\n";

	###  Create a hash of arrays, to index Hit objects by score
	###  Cannot use a simple hash because multiple Hits may have
	###   the same score.
	my %hitscores;
	foreach my $hit (@hits) {
		my $score = $hit->score();
		unless ( $hitscores{$score} ) {
			$hitscores{$score} = [];
		}
		my $ref  = $hitscores{$score};
		my @list = @$ref;
		push @list, $hit;
		@$ref = @list;
	}

	### Sort the Hit objects into descending order of scores
	my @unordered_scores = keys(%hitscores);
	my @ordered_scores = sort { $b <=> $a } @unordered_scores;
	my @ordered_hits;
	foreach my $score (@ordered_scores) {
		my $ref  = $hitscores{$score};
		my @hits = @$ref;
		foreach my $hit (@hits) {
			push @ordered_hits, $hit;
		}
	}
	### Print out number of hits
	print "Num of Hits|".scalar(@ordered_hits)."\n";


	### Now print out the hits ...
	foreach my $hit (@ordered_hits) {
		my ( $pos, $str, $seq, $score, $locus ) = (
			$hit->position(), $hit->strand(),
			$hit->sequence(), $hit->score(),
			$hit->locus()
		);
		printf "%8s|%1s|%s|%5d|%s\n", $pos, $str, $seq, $score, $locus;
	}

	### If we have opted to calculate statistics ...
	if ( $option{s} ) {

		### Calculate mean score for all possible windows in the
		### query sequence
		my $total_score = 0;
		for ( my $i = 0 ; $i < $seq_length ; $i++ ) {
			$total_score =
```

```
                        $total_score + $all_scores_forward{$i} + $all_scores_reverse{$i};
                }

                $mean_score = $total_score / ( $seq_length * 2 );
                print "Mean score = $mean_score\n";

                ### Calculate variance and sd of scores for all
                ### possible windows in the query sequence
                $total_dev = 0;
                for ( my $i = 0 ; $i < $seq_length ; $i++ ) {
                        $total_dev =
                          $total_dev + ( ( $all_scores_forward{$i} - $mean_score )**2 ) +
                          ( ( $all_scores_reverse{$i} - $mean_score )**2 );
                }

                $variance = $total_dev / ( $seq_length * 2 );
                print "Variance = ", $variance, "\n\n\n";
                $sd = $variance**(0.5);
        }
        ### print time - $^T;

        $seq_fh->close;

}    # end of promscan_kl()

sub locate_match {
        my $ptt_file          = shift or die "Failed to pass a PTT file";
        my $promoter_position = shift or die "Failed to pass a position\n";
        my $promoter_strand   = shift or die "Failed to pass a strand\n";
        my $sequence          = shift or die "Failed to pass a sequence\n";
        my $seq_window        = shift
          or die
"Filed to pass a seq_window ($ptt_file,$promoter_position,$promoter_strand\n";

        unless ( defined $option{n} ) {

                my ( $start_pos1, $end_pos1, $strand1, $product1 ) =
                  ( 0, 0, "?", "ORIGIN" );
                my ( $start_pos2, $end_pos2, $strand2, $product2 ) =
                  ( 0, 0, "?", "ORIGIN" );

                my $promoter_description = "";
                ### $promoter_description = "not found";
                foreach my $read_line (@$ptt_file) {

                        # Read each window of the protein table file
                        my @fields = read_ptt_line($read_line);

                        unless ( $fields[0] eq "failed" ) {

                                ( $start_pos1, $end_pos1, $strand1, $product1 ) =
                                  ( $start_pos2, $end_pos2, $strand2, $product2 );
                                ( $start_pos2, $end_pos2, $strand2, $product2 ) = @fields;

                                my $size_of_intergenic_region = 0;
                                my $upstream_sequence         = "";
```

```
                ### Check wether promoter falls within this window
                if (   $promoter_position > $end_pos1
                        && $promoter_position < $start_pos2 )
                {

                        ###  promoter is in intergenic region...
                        my $size_of_intergenic_region_temp =
                          $start_pos2 - $end_pos1;

                        if ( $promoter_strand eq "+" && $strand2 eq "+" ) {
                                $size_of_intergenic_region =
                                  $size_of_intergenic_region_temp;
                                $upstream_sequence = substr(
                                        $sequence,
                                        ( $end_pos1 - 50 ),
                                        ( $size_of_intergenic_region + 49 )
                                );

                                my $spacer = $start_pos2 - $promoter_position -
                                  length($seq_window);
                                $promoter_description =
                                  "-N($spacer)- $product2|$size_of_intergenic_region";

                        }

                        elsif ( $promoter_strand eq "-" && $strand1 eq "-" ) {
                                $size_of_intergenic_region =
                                  $size_of_intergenic_region_temp;
                                my $seq_tmp = substr(
                                        $sequence,
                                        ( $end_pos1 - 2 ),
                                        ( $size_of_intergenic_region + 52 )
                                  )
                                  or die
"Failed to get seq_tmp! end_pos1=$end_pos1, size_of_intergenic_region=$size_of_intergenic_region\n";

                                my $spacer = $promoter_position - $end_pos1;
                                $promoter_description =
                                  "-N($spacer)- $product1|$size_of_intergenic_region";

                        }

                        elsif ( $promoter_strand eq "-" && $strand1 eq "+" ) {
                                $size_of_intergenic_region =
                                  $size_of_intergenic_region_temp;
                                my $seq_tmp = substr(
                                        $sequence,
                                        ( $end_pos1 - 2 ),
                                        ( $size_of_intergenic_region + 52 )
                                  )
                                  or die
"Failed to get seq_tmp! end_pos1=$end_pos1, size_of_intergenic_region=$size_of_intergenic_region\n";

                                my $spacer = $promoter_position - $end_pos1;
                                $promoter_description =
```

```
"-N($spacer)- $product1 (WRONG ORIENTATION)|$size_of_intergenic_region";

                                        }

                                        elsif ( $promoter_strand eq "+" && $strand1 eq "-" ) {
                                                $size_of_intergenic_region =
                                                  $size_of_intergenic_region_temp;
                                                my $seq_tmp = substr(
                                                        $sequence,
                                                        ( $end_pos1 - 2 ),
                                                        ( $size_of_intergenic_region + 52 )
                                                  )
                                                  or die
"Failed to get seq_tmp! end_pos1=$end_pos1, size_of_intergenic_region=$size_of_intergenic_region\n";

                                                my $spacer = $promoter_position - $end_pos1;
                                                $promoter_description =
"-N($spacer)- $product1 (WRONG ORIENTATION)|$size_of_intergenic_region";

                                        }
                                }

                                elsif ( ( $promoter_position >= $start_pos1 )
                                        and ( $promoter_position <= $end_pos1 )
                                        and ( $option{i} ) )
                                {
                                        $promoter_description = "internal to $product1";
                                }
                        }

                }
                my $size_of_intergenic_region = $start_pos2 - $end_pos1;
                return ( $promoter_description, $size_of_intergenic_region );

        }
}    # End of sub locate_match

sub read_ptt_line {

        # Expects a scalar value containing one line of a .ptt file
        # Extracts the 'location', strand, and 'product'
        #   from that line and returns them, in that order

        my $read_line = $_[0];
        my $i;
        my $product;
        my @fields;

        if ( $read_line =~ /(\d+)\.\.(\d+)\s+([+-])/ ) {

                # Extract 'location' and 'strand'
                @fields  = split( /\s+/, $read_line );
                $i       = 5;
                $product = "";
                while ( $fields[$i] ) {

```

```
					# Extract 'product'
					$product = $product . " " . $fields[$i];
					$i++;
				}
				return ( $1, $2, $3, $product );
			}
			;	# End of 'if( $read_line =~ ....' block

			### print ("variable read_line:$read_line\n");
			return ("failed");

};	# end of sub read_ptt_line;

sub calculate_GC {
		my (@sequence) = @_;
		my ( $n, $GC, $AT ) = ( 0, 0, 0 );	# initialise counters
		my $seqread;

		foreach my $seqread (@sequence) {
				$GC++ if $seqread =~ /[GCgc]/;
				$AT++ if $seqread =~ /[ATat]/;
		}
		$n = $GC + $AT;
		my $GC_content = $GC / $n;
		return $GC_content;
}

sub revcomp {
		my $string = shift @_ or print STDERR "Called revcomp on null string!";
		my $seq    = "";
		my @array  = split( //, $string );
		while ( my $char = pop @array ) {
				$char =~ tr/ACGT/TGCA/;
				$seq = $seq . $char;
		}

		#print STDERR "Revcomp of $string -> $seq\n" ;
		return $seq;
}

sub read_matrix_file {

		my $matrix_file = shift;
		my %matrix;

		my $fh = new IO::File;
		$fh->open("<$matrix_file")
		  and print STDERR "Opened matrix file: $matrix_file\n"
		  or die "Could not open Configuration file $matrix_file: $!\n";

		### print "\nScoring matrix:\n\n";

		while ( my $readline = <$fh> ) {
				chomp($readline);

				if ( $readline =~ m/([ACGT]):([\s\d]+)/ ) {
```

```
			###	print "$readline\n";
				print STDERR "$readline\n";

				my $base   = $1;
				my @values = split /\s+/, $2;

				$matrix{$base} = [];
				foreach my $value (@values) {
					push @{ $matrix{$base} }, $value;
				}
			}
		}

###	print "\n\n";
	print STDERR "\n\n";

	$fh->close;
	print STDERR "Closed matrix file\n";

	return \%matrix;
}

sub show_help {
	print STDERR <<EOF;


=================================================================================
==================
$0: scan a DNA sequence against a motif specified as a scoring matrix
=================================================================================
==================

Usage: $0 <options>

   Available options

       -h           : Show this help.
            -l           : Use linear scoring formula ignoring G+C content,
                    rather than the Kullback-Leibler distance.
       -s           : Calculate statistics (this requires a lot of memory!)
       -i           : Show matches that are internal to ORFs as well as intergenic ones.
       -n           : Do not use a .ptt file (ie no ORF information)
            -g <file>     : Specify the input file (genome)
                    You can specify the .ppt file or the .fna file
                    or just the base name:
                    eg. $0 -g NC_000962.ptt
                       $0 -g NC_000962.fna
                       $0 -g NC_000962
            -m <file>     : Specify the matrix file.
            -c <number>   : Specify the threshold (cut-off) score (default is 1000).
                    You may wish to experiment with various threshold values
                     to get the optimum balance between sensitivity and accuracy.

=================================================================================
===================

```

```
Output is printed to the output file as well as to STDOUT (ie the screen).
For each hit, ie a close match to the consensus sequence that is being sought,
the output is arranged into six columns:

Column 1		: The position of the hit in the input sequence.
Column 2		: The strand on which the hit is found (+/-).
Column 3		: Sequence of the hit.
Column 4		: A score for the hit.  The higher the score, the better the match!
Column 5		: The position of the hit relative to adjacent/downstream Open Reading Frames
Column 6		: The size of the intergenic region in which the match is found.

For more information see the PromScan website http://www.promscan.uklinux.net

================================================================================
====================
EOF
}

### Hit.pm class of objects representing a hit,
###  i.e. a good match to the sought sequence
###  defined by the frequency matrix

package Hit;

sub new {
	my $self = {};
	bless $self, "Hit";
	return $self;
}

sub position {
	my $self = shift @_;
	if ( my $specified = shift @_ ) {
		$$self{POSITION} = $specified;
	}
	return $$self{POSITION};
}

sub strand {
	my $self = shift @_;
	if ( my $specified = shift @_ ) {
		$$self{STRAND} = $specified;
	}
	return $$self{STRAND};
}

sub score {
	my $self = shift @_;
	if ( my $specified = shift @_ ) {
		$$self{SCORE} = $specified;
	}
	return $$self{SCORE};
}

sub sequence {
	my $self = shift @_;
```

```
		if ( my $specified = shift @_ ) {
			$$self{SEQ} = $specified;
		}
		return $$self{SEQ};
}

sub locus {
		my $self = shift @_;
		if ( my $specified = shift @_ ) {
			$$self{LOCUS} = $specified;
		}
		return $$self{LOCUS};
}

sub intergenic_size {
		my $self = shift @_;
		if ( my $specified = shift @_ ) {
			$$self{SIZE} = $specified;
		}
		return $$self{SIZE};
}

return 1;	# end of package
```
