# Supplementary material for: Alternative Sigma Factor Over-Expression Enables Heterologous Expression of a Type II Polyketide Biosynthetic Pathway in Escherichia coli
Source: PLoS One. 2013 May 28;8(5):e64858. doi: 10.1371/journal.pone.0064858 (PMC3665592; doi:10.1371/journal.pone.0064858)
Supplement: Table S1 — Complete data set for qPCR analysis as shown in Figures 1 and 3 . Standard curve, efficiencies, R2 and Ct values are given. (PDF) [file pone.0064858.s006.pdf]

| Strain and Std Curve | *Gene* | Ct (dRn) |
|---|---|---|
| **BAP1+DCS11**<br>Y = -3.245*LOG(X) + 18.68; Eff.= 103.6%; $R^2$=0.991 | | |
| | *rpoN* | 24.19 |
| | | 20.95 |
| | | 18.24 |
| | *oxyB* | 38.87 |
| | | 38.66 |
| | | 39.66 |
| | *oxyF* | 31.65 |
| | | 31.7 |
| | | 31.94 |
| | *oxyK* | 32.18 |
| | | 29.07 |
| | | 31.04 |
| | *oxyP* | 33.97 |
| | | 35.06 |
| | | 34.33 |
| | *oxyT* | 33.95 |
| | | 34.39 |
| | | 37 |
| **BAP1+MRH08**<br>Y = -3.321*LOG(X) + 14.72; Eff.= 100.0%; $R^2$=0.992 | | |
| | *rpoN* | 19.79 |
| | | 23.11 |
| | | 21.11 |
| | *oxyB* | No Ct |
| | | 35.13 |
| | | 31.25 |
| | *oxyF* | 20.17 |
| | | 23.97 |
| | | 22.63 |
| | *oxyK* | 18.02 |
| | | 22.79 |
| | | 17.12 |
| | *oxyP* | 19 |
| | | 24.6 |
| | | 20.2 |
| | *oxyT* | 23 |
| | | No Ct |
| | | 25.25 |
| **BAP1+MRH08+DCS11**<br>Y = -3.245*LOG(X) + 18.68; Eff.= 103.6%; $R^2$=0.991 | | |
| | *rpoN* | 15.9 |
| | | 15.86 |
| | | 15.15 |
| | *oxyB* | 26.19 |

| | | |
|---|---|---|
| | | 23.92 |
| | *oxyF* | 24.41 |
| | | 25.79 |
| | *oxyK* | 21.2 |
| | | 22.06 |
| | *oxyP* | 22.43 |
| | | 23.77 |
| | *oxyT* | 29.07 |
| | | 27.99 |
| **BAP1+MRH08+DCS57**<br>Y = -3.313*LOG(X) + 22.40; Eff. = 100.5%; $R^2$=0.997 | | |
| | *oxyB* | 39.53 |
| | | 39.47 |
| | | 30.66 |
| | *oxyK* | 29.4 |
| | | 32.1 |
| | | 28.6 |
| BAP1+MRH08+DCS58<br>Y = -3.364*LOG(X) + 24.24; Eff. = 98.5%; $R^2$=0.997 | | |
| | *oxyB* | No Ct |
| | | No Ct |
| | | 32.73 |
| | *oxyK* | 26.89 |
| | | 26.28 |
| | | 32.57 |
| **BAP1+MRH08+NDP6**<br>Y = -3.27*LOG(X) + 18.29; Eff. = 102.2%; $R^2$=1.00 | | |
| | *oxyB* | 35 |
| | | 27.19 |
| | | 30.43 |
| | *oxyK* | 23.31 |
| | | 24.73 |
| | | 23.33 |
| **BAP1+MRH08+NDP7**<br>Y = -3.145*LOG(X) + 16.20; Eff. = 108.3%; $R^2$=0.995 | | |
| | *oxyB* | 37.35 |
| | | 34.11 |
| | | No Ct |
| | *oxyK* | 22.49 |
| | | 29.45 |
| | | 21.72 |
| **BAP1+MRH08+DCS59**<br>Y = -3.245*LOG(X) + 18.68; Eff.= 103.6%; $R^2$=0.991 | | |
| | *oxyB* | 34.61 |
| | | 23.63 |
| | | 27.26 |
| | *oxyK* | 25.19 |

| | | |
|---|---|---|
| | | 21.98 |
| | | 22.54 |
| **BAP1+DCS61+DCS11**<br>Y = -2.598*LOG(X) + 23.66; Eff. = 142.6%; $R^2$=0.974 | | |
| | *oxyB* | 26.35 |
| | | 26.95 |
| **BAP1+DCS62+DCS11**<br>Y = -2.621*LOG(X) + 23.80; Eff. = 140.7%; $R^2$=0.979 | | |
| | *oxyB* | 29.95 |
| | | 35.29 |
