# Supplementary material for: Alternative Sigma Factor Over-Expression Enables Heterologous Expression of a Type II Polyketide Biosynthetic Pathway in Escherichia coli
Source: PLoS One. 2013 May 28;8(5):e64858. doi: 10.1371/journal.pone.0064858 (PMC3665592; doi:10.1371/journal.pone.0064858)
Supplement: Table S3 — List of PKS, NRPS and NRPS/PKS gene clusters with the associated σ54 promoters identified from our bioinformatics analysis. (PDF) [file pone.0064858.s008.pdf]

NC_002755.2 Mycobacterium tuberculosis CDC1551, complete genome

**1**	NRPS	105314-123821						
	Score	Distance From ORF	N	Strand	Sequence	Gene	Synonym	Protein Name
	75	430	106294	+	TTGGCAGCGCTTCGGCT	-	MT0106	dioxygenase, putative
	75	157	106567	+	CTGGCAGTCAAGGTGCC	-	MT0106	dioxygenase, putative
	81	323	109450	+	GTGGCGTGAACATTGCG	-	MT0109	hypothetical protein
	81	325	122488	-	GTGGAATAGCACTTGCC	-	MT0112	cation transporter E1-E2 family ATPase
	81	60	122247	+	ATGGCCCGCTGGATGCA	-	MT0113	hypothetical protein
**2**	PKS	485426-491388						
**3**	PKS	1313164-1328792						
	Score	Distance From ORF	N	Strand	Sequence	Gene	Synonym	Protein Name
	78	341	1327340	-	CTGGCTCAGACCCTGCG	-	MT1222	acyl-CoA synthetase
**4**	PKS	1865066-1886258						
	Score	Distance From ORF	N	Strand	Sequence	Gene	Synonym	Protein Name
	78	121	1866089	+	CTGGCACTGATGCTGGA	-	MT1701	polyketide synthase
	76	370	1878562	+	TTGCCACCGACCTTGAT	-	MT1704	polyketide synthase
**5**	PKS	2294295-2309319						
**6**	NRPS/PKS	2647576-2673168						
	Score	Distance From ORF	N	Strand	Sequence	Gene	Synonym	Protein Name
	75	344	2649233	-	CCGGCCCCGCCGTTGCC	-	MT2439	hypothetical protein
	83	331	2651649	-	GAGGAACGCCTTTTGCT	dnaJ-2	MT2442	chaperone protein DnaJ
	78	334	2673502	-	CTGGCAGCCGTTTGGCA	pchE	MT2451	dihydroaeruginoic acid synthetase
**7**	PKS	3236513-3319822						
	Score	Distance From ORF	N	Strand	Sequence	Gene	Synonym	Protein Name
	76	385	3236128	+	TCGGCACGCCTCGGGCT	-	MT2998	thioesterase
	75	1	3237290	+	TTCGCTGGACATTTGCT	-	MT2998.1	hypothetical protein
	77	349	3256220	+	CTGGGACTCGACTCGCT	-	MT3004	polyketide synthase
	78	23	3292184	-	GTGGCCGACCGTTTGCG	-	MT3021.1	polyketide synthase
	77	323	3296988	-	ATCGCACGGCGCTGGCG	-	MT3023	acyl-CoA synthetase
	79	490	3298272	+	TTGGCATCGATCTTGGG	-	MT3026	methyltransferase, putative
	78	477	3298285	+	TGGGCACGAATTTCGCC	-	MT3026	methyltransferase, putative
	77	104	3298658	+	CTGCCATACTCCTTGCC	-	MT3026	methyltransferase, putative
	82	353	3303494	+	GTGGCATGCTCATTCCT	-	MT3031	glycosyl transferase
	76	44	3303803	+	GTGGCTGCGGTATTGCC	-	MT3031	glycosyl transferase
	79	276	3306596	-	CTGGCAAGATCTTCGCC	-	MT3034	UDP-glucoronosyl and UDP-glucosyltransferase family protein
	75	379	3308002	-	TTGGCCAGCTAGTTACT	-	MT3036	hypothetical protein
	76	106	3308155	-	GTGGCAGGGGCCGTGAT	-	MT3037	hypothetical protein
	89	439	3311348	+	CTGGCACGCTACATGCA	-	MT3041.1	hypothetical protein

NC_008611.1 Mycobacterium ulcerans Agy99, complete genome

**1**	PKS	1803923-1835953						
	Score	Distance From ORF	N	Strand	Sequence	Gene	Synonym	Protein Name
	77	280	1820828	+	CTGGACAAGTTATTGCT	pks11	MUL_1656	chalcone synthase, Pks11

	Score	Distance From ORF	N	Strand	Sequence	Gene	Synonym	Protein Name
	75	91	1821017	+	ATGTCACATGTGTTGAA	pks11	MUL_1656	chalcone synthase, Pks11
	80	234	1834666	-	ATGGTGCCGCAGTTGCT	-	MUL_1664	hypothetical protein
	75	443	1834875	-	ACGGCACAGCATTTTCG	-	MUL_1664	hypothetical protein
	75	322	1835436	-	CTGGATCCCGTTTTGCG	-	MUL_1665	antibiotic resistance ABC transporter, efflux protein
**2**	PKS		2211634-2271101					
	Score	Distance From ORF	N	Strand	Sequence	Gene	Synonym	Protein Name
	75	133	2213368	+	CGGGCACACCGAGTGCG	-	MUL_2003	chorismate pyruvate-lyase
	78	133	2216250	+	GTGGCCGAACGGTTGCG	pks15/1	MUL_2005	polyketide synthase Pks15/1
	79	305	2228469	+	GTGGCTCAACGGTGGCT	-	MUL_2009	methyltransferase
	75	67	2230104	+	GTGTCACACCGGTTGAA	mas	MUL_2010	multifunctional mycocerosic acid synthase membrane-associated Mas
	78	354	2240002	-	ACGGCAAGATCATTGCC	drrB	MUL_2013	daunorubicin-DIM-transport integral membrane protein ABC transporter DrrB
	76	153	2240793	-	CTGGCGCGGTCGTTCCC	drrA	MUL_2014	daunorubicin-DIM-transport ATP-binding protein ABC transporter DrrA
	77	288	2240928	-	TTGGCAATGCCGGTGCG	drrA	MUL_2014	daunorubicin-DIM-transport ATP-binding protein ABC transporter DrrA
	84	178	2245274	-	GTGACACTGATTTTGCA	ppsE	MUL_2015	phenolpthiocerol synthesis type-I polyketide synthase PpsE
	80	247	2250769	-	ATGGCCCTCGAGTTGCG	ppsD	MUL_2016	phenolpthiocerol synthesis type-I polyketide synthase PpsD
	82	34	2257161	-	TTGGAACAGCTTTCGCA	ppsC	MUL_2017	phenolpthiocerol synthesis type-I polyketide synthase PpsC
	76	121	2266630	-	GTGGCCGACGTCGTGCT	ppsA	MUL_2019	phenolpthiocerol synthesis type-I polyketide synthase PpsA
**3**	NRPS		2921595-2959529					
	Score	Distance From ORF	N	Strand	Sequence	Gene	Synonym	Protein Name
	79	76	2921519	+	TCGGCACGATAATTCCT	-	MUL_2620	hypothetical protein
	80	216	2929670	-	ATGTCGCGGGCATTGCT	-	MUL_2625	hypothetical protein
	81	92	2930909	-	CTGGCGCAGACCGTGCA	fadE25	MUL_2626	acyl-CoA dehydrogenase FadE25
	75	87	2934800	-	GTCGCGCGGGGATTGCC	birA	MUL_2631	bifunctional protein BirA
	76	286	2952542	-	GTGGCAGACCTGCGGCT	sigF	MUL_2640	RNA polymerase sigma factor SigF
	83	205	2953689	-	GTGGCACGGCGCCGGCT	usfY	MUL_2642	hypothetical protein
**4**	NRPS/PKS		4029686-4059363					
	Score	Distance From ORF	N	Strand	Sequence	Gene	Synonym	Protein Name
	76	385	4033562	-	TTGGACTACGGCTTGCT	mbtB	MUL_3632	phenyloxazoline synthase MbtB
	75	397	4038148	-	CTGGCCCCACCGCTGCC	mbtF	MUL_3633	non-ribosomal peptide synthetase MbtF
	75	61	4042974	+	GTTGAATGGCTGTTGCG	-	MUL_3635	hypothetical protein
	76	245	4044260	+	CTGGCACTTCGCGTGAT	mbtC	MUL_3637	polyketide synthase MbtC
	76	56	4050456	-	ATGGCAACGCTATTACC	mbtG	MUL_3640	lysine-N-oxygenase MbtG
	80	31	4053325	+	ATCGCAGGCTCATTGCA	-	MUL_3643	short-chain membrane-associated dehydrogenase
	75	317	4057245	-	CTGGCTCGGCAGCGGCA	desA1_1	MUL_3646	acyl-[acyl-carrier protein] desaturase DesA1_1

**5**	NRPS	4833556-4838804						
**6**	NRPS	5377263-5405727						
	Score	Distance From ORF	N	Strand	Sequence	Gene	Synonym	Protein Name
	77	255	5378620	+	ACGGCACCAGCACTGCT	-	MUL_4854	oxidoreductase
	75	467	5379274	+	CTGGCCAAGGTTTGGCA	-	MUL_4855	hypothetical protein
	84	398	5391216	-	TCGGCCCGGATATTGCT	-	MUL_4861	hypothetical protein
	83	199	5396076	-	ATGGCAATGACGTTGCG	-	MUL_4867	hypothetical protein
**7**	PKS	5523988-5532849						

NC_006361.1 Nocardia farcinica IFM 10152, complete genome

**1**	PKS	184410-208907						
	Score	Distance From ORF	N	Strand	Sequence	Gene	Synonym	Protein Name
	78	160	207612	+	GTGGCGCCGTCGGTGCT	-	nfa1980	hypothetical protein
**2**	NRPS	742402-783087						
	Score	Distance From ORF	N	Strand	Sequence	Gene	Synonym	Protein Name
	78	5	782060	+	GTGGCACGCGTGCCGCG	-	nfa7210	putative esterase
**3**	NRPS/PKS	809229-843740						
	Score	Distance From ORF	N	Strand	Sequence	Gene	Synonym	Protein Name
	75	426	815841	-	GTGGGCCAACAACTGCT	-	nfa7550	putative ATP-dependent protease
	78	324	816789	-	CTGGCCTGGGCGGTGCT	amiE	nfa7560	acylamide amidohydrolase
	78	239	820253	-	TTGGCGCAACACGTGCG	-	nfa7600	hypothetical protein
	80	307	820321	-	CTGGCGAGCCAGTTGCC	-	nfa7600	hypothetical protein
	76	231	827277	+	CTGGCCGAGCGGATGCT	nbtD	nfa7660	putative non-ribosomal peptide synthetase
	76	64	837122	+	CTGGCCGAGCTGTGGCA	nbtF	nfa7680	putative non-ribosomal peptide synthetase
**4**	NRPS	1219367-1235955						
	Score	Distance From ORF	N	Strand	Sequence	Gene	Synonym	Protein Name
	77	475	1218892	+	CTGGCCCGTTGCCTGCG	-	nfa11040	hypothetical protein
**5**	NRPS	2965856-2985159						
**6**	PKS	3189119-3223839						
	Score	Distance From ORF	N	Strand	Sequence	Gene	Synonym	Protein Name
	76	247	3191679	-	CTGGCGCTGGTGCTGCG	-	nfa30090	putative iron-sulfur oxidoreductase
	75	103	3193577	-	CTAGCGCGCGGATTGCG	-	nfa30110	putative monooxygenase
	75	380	3193854	-	GTGGCATCCTGGTGGCC	-	nfa30110	putative monooxygenase
	76	406	3195498	-	CTGGGGCAGTGATTGCG	-	nfa30120	acyl-CoA synthetase
	75	62	3198518	+	CCGGCAAGCGCGATGCT	-	nfa30170	hypothetical protein
	76	56	3202150	+	ATTGCTGGAATATTGCT	-	nfa30220	putative sigma factor
	75	459	3212616	-	CTGGCGGACCAACTGCT	-	nfa30250	putative polyketide synthase
	76	221	3220860	-	CTGGACTTCCTGTTGCT	-	nfa30320	hypothetical protein
**7**	NRPS	3297940-3312573						
	Score	Distance From ORF	N	Strand	Sequence	Gene	Synonym	Protein Name

Score	Distance From ORF	N	Strand	Sequence	Gene	Synonym	Protein Name
78	179	3299108	-	GTGGCCGGGACCGTGCA	-	nfa31160	putative ABC transporter ATP-binding protein
77	296	3299225	-	CTGGCGCGGGACTTCCG	-	nfa31160	putative ABC transporter ATP-binding protein

8 PKS 4478704-4491458

Score	Distance From ORF	N	Strand	Sequence	Gene	Synonym	Protein Name
76	338	4489377	-	ATGGCACAGGTGGCGCC	-	nfa43240	putative polyketide synthase
77	493	4491951	-	CCGGCAACGCACTTGCC	-	nfa43260	putative transporter

9 NRPS 5244714-5291138

10 NRPS 5318160-5361768

11 PKS 5919882-5934805

Score	Distance From ORF	N	Strand	Sequence	Gene	Synonym	Protein Name
80	97	5923205	+	GCGGCACGTGAGCTGCT	-	nfa55870	hypothetical protein
79	153	5926963	-	GTGGCACGACCGTGCCA	-	nfa55900	long-chain-fatty-acid--CoA ligase
78	189	5926962	+	ATGGCACGGTCGTGCCA	-	nfa55910	putative dioxygenase

NC_009142.1 *Saccharopolyspora erythraea* NRRL 2338, complete genome

1 PKS 14813-51921

Score	Distance From ORF	N	Strand	Sequence	Gene	Synonym	Protein Name
81	175	15677	-	TTGGCCGGGCCGTTGCG	-	SACE_0012	TetR family transcriptional regulator
76	129	49054	+	GCGGCCCGGCTCTTTCT	-	SACE_0029	RNA polymerase sigma factor

2 PKS 778214-832825

Score	Distance From ORF	N	Strand	Sequence	Gene	Synonym	Protein Name
76	328	783452	-	CTGGCGCCTACGGTGCA	eryCIV	SACE_0716	eryCIV NDP-6-deoxyhexose 3,4-dehydratase
76	128	784712	-	CTGGCACAGGTGATCCG	eryBVI	SACE_0717	NDP-4-keto-6-deoxy-glucose 2,3-dehydratase
77	362	787113	-	GTGGCACGACGGCGGCG	eryBV	SACE_0719	6-DEB TDP-mycarosyl glycosyltransferase
81	224	831762	-	GAGGTACGCGGCTTGCA	ermE	SACE_0733	N-6-aminoadenine-N-methyltransferase,erythromycin resistance

3 NRPS 1436967-1445722

4 PKS 2528343-2549947

Score	Distance From ORF	N	Strand	Sequence	Gene	Synonym	Protein Name
76	242	2528101	+	GTGGACCGGCAGTTGGT	cmtC	SACE_2339	trehalose corynomycolyl transferase C

5 PKS 2795362-2818679

Score	Distance From ORF	N	Strand	Sequence	Gene	Synonym	Protein Name
76	132	2798967	-	TCGGCACGCGACTTGGG	-	SACE_2592	glucose-methanol-choline oxidoreductase
80	116	2800190	-	CTGGCGCACCGGGTGCT	-	SACE_2593	glycopeptide antibiotics resistance protein

6 NRPS/PKS 2838066-2886848

	Score	Distance From ORF	N	Strand	Sequence	Gene	Synonym	Protein Name
	81	433	2844648	-	GTGGCGCAGGTGCTGCA	-	SACE_2619	non-ribosomal peptide synthetase
	79	322	2855647	-	GAGGCACTGCGCCTGCT	-	SACE_2627	isopentenyl-diphosphate delta-isomerase II 2
	77	316	2883545	-	GTGGCGCTGGCGCTGCA	-	SACE_2635	major facilitator superfamily transporter phthalate permease
**7**	NRPS	2944491-2974637						
	Score	Distance From ORF	N	Strand	Sequence	Gene	Synonym	Protein Name
	75	112	2962692	+	GTGGAACGGCCGCGGCA	-	SACE_2698	putative regulatory protein
	76	63	2974297	+	GTGGCACGCGCGGCGCC	-	SACE_2709	hypothetical protein
**8**	PKS	3127652-3157229						
	Score	Distance From ORF	N	Strand	Sequence	Gene	Synonym	Protein Name
	76	284	3131486	-	TCGGCACGGTGTTCGCC	rifI	SACE_2867	aminoquinate/shkimate dehydrogenase
	79	195	3138945	+	GTGCCATTCCGGTTGCT	-	SACE_2875	modular polyketide synthase
	77	158	3138982	+	CAGGCATCGAGGTTGCG	-	SACE_2875	modular polyketide synthase
	78	428	3151884	+	CTGGCGCACGTGTTCCT	mhpA	SACE_2877	3-(3-hydroxyphenyl)propionate hydroxylase
	77	240	3152072	+	CTGGCACGGCCTCTACC	mhpA	SACE_2877	3-(3-hydroxyphenyl)propionate hydroxylase
**9**	NRPS	3301269-3308199						
	Score	Distance From ORF	N	Strand	Sequence	Gene	Synonym	Protein Name
	77	55	3307140	+	ACGGCGCGGATCGTGCT	-	SACE_3016	SyrP-like protein
**10**	NRPS	3326161-3344964						
	Score	Distance From ORF	N	Strand	Sequence	Gene	Synonym	Protein Name
	85	242	3325919	+	GTGGCCAGGCGGTTGCT	-	SACE_3033	putative peptide monooxygenase
**11**	PKS	4545937-4616845						
	Score	Distance From ORF	N	Strand	Sequence	Gene	Synonym	Protein Name
	75	59	4554457	+	TTGACTAGAGTGTTGCT	-	SACE_4130	hypothetical protein
	78	290	4559173	+	ATGGCCTCGCGGTTGCC	-	SACE_4132	acyl carrier protein
	86	49	4563179	+	TGGGCACCGCCCTTGCT	-	SACE_4135	MaoC-like dehydratase
	80	259	4572145	-	CTGGCCGGGCACCTGCT	-	SACE_4138	type I PKS modular polyketide synthas
	78	417	4588385	-	GTGGAACAGGCCTTCCA	-	SACE_4139	type I modular polyketide synthase
	75	146	4612861	-	GTGGCGCTGCGCGTGCC	-	SACE_4142	cytochrome P450 monooxygenase
	75	251	4612966	-	CTGGCTCGGATGGAGCT	-	SACE_4142	cytochrome P450 monooxygenase
	83	27	4614155	-	ATGGCAGGGAAGTTCCT	-	SACE_4143	cytochrome P450 monooxygenase
**12**	NRPS	4759004-4809694						
	Score	Distance From ORF	N	Strand	Sequence	Gene	Synonym	Protein Name
	78	475	4772132	-	CTGGCCCGGGCGCTGCG	-	SACE_4285	AMP-dependent synthetase and ligase

	Score	Distance From ORF	N	Strand	Sequence	Gene	Synonym	Protein Name
	76	498	4772155	-	GTGGCGCGCTGCTGGCC	-	SACE_4285	AMP-dependent synthetase and ligase
**13**	PKS	4821866-4828194						
**14**	PKS	4992823-5006652						
	Score	Distance From ORF	N	Strand	Sequence	Gene	Synonym	Protein Name
	75	183	4996606	-	GTGGACCGCCCGGTGCA	-	SACE_4472	NAD-dependent epimerase/dehydratase
	79	82	4997888	-	GTGGCGCTGAAGCTGCT	qor	SACE_4474	quinone oxidoreductase
**15**	PKS	5102367-5117363						
	Score	Distance From ORF	N	Strand	Sequence	Gene	Synonym	Protein Name
	79	327	5106524	+	CTCGCACGCGGCCTGCA	-	SACE_4566	hypothetical protein
	78	359	5112790	+	GTGCCGCAACGTTTGCT	pteA1	SACE_4574	modular polyketide synthase
**16**	PKS	5932907-5941187						
	Score	Distance From ORF	N	Strand	Sequence	Gene	Synonym	Protein Name
	78	213	5933803	+	CGGCCACGCCGGTTGCA	chlB1	SACE_5308	iterative type I polyketide synthase
**17**	PKS	6225358-6248235						
	Score	Distance From ORF	N	Strand	Sequence	Gene	Synonym	Protein Name
	78	287	6239374	-	TCGGCACCGCTCTTCCA	-	SACE_5540	3-oxoacyl (acyl carrier protein) synthase III

NC_009380.1 Salinispora tropica CNB-440, complete genome

	Score	Distance From ORF	N	Strand	Sequence	Gene	Synonym	Protein Name
**1**	PKS	671414-679577						
	Score	Distance From ORF	N	Strand	Sequence	Gene	Synonym	Protein Name
	75	214	671873	+	TTGGAACGAAACGAGCT	-	Strop_0597	hypothetical protein
	83	374	672939	+	ATGGCTGGCACGTTGCT	-	Strop_0598	beta-ketoacyl synthase
**2**	NRPS/PKS	144505-1172682						
	Score	Distance From ORF	N	Strand	Sequence	Gene	Synonym	Protein Name
	79	296	1150310	-	CTGGCATGCCTCCGGCA	-	Strop_1020	MbtH domain-containing protein
	82	296	1164400	+	GTGGCATGGCCTATGCC	-	Strop_1028	hypothetical protein
	76	157	1164539	+	CTGGCCGGCCGATCGCT	-	Strop_1028	hypothetical protein
	78	220	1165260	+	CTGCCACGGCTGCTGCC	-	Strop_1029	cyclase family protein
	81	438	1167460	-	CTGGCACAGATAGCGCT	-	Strop_1030	regulatory protein, LuxR
**3**	PKS	2502319-2520690						
	Score	Distance From ORF	N	Strand	Sequence	Gene	Synonym	Protein Name
	76	33	2505275	-	TGGGCACAAAGCCTGCG	-	Strop_2212	TAP domain-containing protein
	75	447	2507023	-	GCGGCACCCTGGTTGAT	-	Strop_2213	hypothetical protein
	78	480	2513894	-	ATGGGACTGACCTCGCT	-	Strop_2220	hypothetical protein
	83	411	2514406	+	CTGGTAGAGCAGTTGCA	-	Strop_2222	dTDP-glucose 4,6-dehydratase
	75	1	2517088	+	TGGGCACGGGTCCGGCA	-	Strop_2224	beta-ketoacyl synthase
	76	463	2519253	+	GCGGCCGGCCCGTTGCT	-	Strop_2227	hypothetical protein
**4**	PKS	2795012-2816666						
	Score	Distance From ORF	N	Strand	Sequence	Gene	Synonym	Protein Name

Score	Distance From ORF	N	Strand	Sequence	Gene	Synonym	Protein Name
75	433	2798754	-	CTGGTCGAACTCTTGCG	-	Strop_2487	dehydrogenase, E1 component
76	124	2804213	-	GAGGCCGACCTGTTGCT	-	Strop_2492	AMP-dependent synthetase and ligase
81	84	2805809	-	CTGGCCTGGCTTGTGCA	-	Strop_2494	beta-ketoacyl synthase
80	205	2809136	-	GTGGCGACCGTGTTGCT	-	Strop_2498	antibiotic biosynthesis monooxygenase
77	357	2812320	-	GTGGCCGAGATCCTGCA	-	Strop_2501	cupin 2 domain-containing protein
77	112	2814752	-	CTGGCGCACCTGGTGCC	-	Strop_2504	acetyl-CoA carboxylase, biotin carboxyl carrier protein

5	NRPS/PKS	2956815-2996840						
	Score	Distance From ORF	N	Strand	Sequence	Gene	Synonym	Protein Name
	78	393	2974666	-	TTGGCGCAGCTCTGGCC	-	Strop_2648	methyltransferase type 12
	80	85	2977211	-	CTGGCACCCGCCGTGCC	-	Strop_2650	ABC transporter related
	75	466	2979339	-	CTGGCGTTGACCCTGCA	-	Strop_2652	binding-protein-dependent transport systems inner membrane component
	77	150	2996990	-	TTGGCCAAGCAAGTGCT	-	Strop_2659	hypothetical protein

6	PKS	3020222-3042851						
	Score	Distance From ORF	N	Strand	Sequence	Gene	Synonym	Protein Name
	76	204	3023263	-	GTGGCCTGGTTCTGGCC	-	Strop_2686	alpha amylase, catalytic region
	75	322	3025145	-	TTGGCATGTGGTGCGCT	-	Strop_2688	helix-turn-helix domain-containing protein
	75	330	3026030	-	GCGGCACCGGCGTTGTT	-	Strop_2689	hypothetical protein
	76	207	3027623	-	ACGGCCCGTCTCGTGCT	-	Strop_2691	hypothetical protein
	81	377	3031869	-	TTGGCAGGGCCGGTGCC	-	Strop_2695	flavin reductase domain-containing protein
	77	192	3037925	-	CTGGCGCTGGCCTCGCA	-	Strop_2697	beta-ketoacyl synthase
	76	235	3043086	-	ATGGCACGGGTGTTCTC	-	Strop_2701	hypothetical protein

7	NRPS/PKS	3112454-3249126						
	Score	Distance From ORF	N	Strand	Sequence	Gene	Synonym	Protein Name
	75	422	3116359	+	ACGGCATGAGCGTCGCA	-	Strop_2766	regulatory protein, TetR
	75	117	3141159	-	CTGGTCCGGCTGGTGCC	-	Strop_2770	cytochrome P450
	80	282	3142490	+	CTGGCCCGGACCGTGCG	-	Strop_2772	aminotransferase, class I and II
	76	440	3143585	+	CTGGCCGGCCCGATGCA	-	Strop_2773	hypothetical protein
	76	344	3143681	+	GTGGCCGGCGCCGTGCA	-	Strop_2773	hypothetical protein
	77	410	3144589	+	TTGGCGCAGCGGCTGCG	-	Strop_2774	AMP-dependent synthetase and ligase
	79	25	3160851	-	CTGGCAACGAGTTTGGT	-	Strop_2778	beta-ketoacyl synthase
	85	327	3165966	-	CTGGCCCGGCACCTGCT	-	Strop_2779	acyl transferase domain-containing protein
	76	471	3188122	-	GCGGCACGCCGATCGCC	-	Strop_2785	hypothetical protein
	77	312	3190952	-	AGGGCAAGCGGATTGCC	-	Strop_2787	AMP-dependent synthetase and ligase
	75	112	3193950	-	CTGGCCGCGACCCTGCT	-	Strop_2791	alpha/beta hydrolase fold
	75	303	3194141	-	CTGGCCGACCCGCTGCT	-	Strop_2791	alpha/beta hydrolase fold
	78	427	3198676	-	TTGGCACTCTCATGGCC	-	Strop_2795	beta-ketoacyl synthase
	78	168	3198676	-	TTGGCACTCTCATGGCC	-	Strop_2796	phosphopantetheine-binding
	79	373	3198881	-	GCGGCACTGCTCTGGCT	-	Strop_2796	phosphopantetheine-binding
	75	470	3198978	-	CGGGAACGAGCCCTGCT	-	Strop_2796	phosphopantetheine-binding
	75	370	3201972	-	CAGGCACGGCTGCGGCA	-	Strop_2800	L-carnitine dehydratase/bile acid-inducible protein F
	76	7	3203132	+	CTGGCACTCTGGAGGCT	-	Strop_2803	AMP-dependent synthetase and ligase

	Score	Distance From ORF	N	Strand	Sequence	Gene	Synonym	Protein Name
	78	466	3212075	+	CTGGCGCTGGAGGTGCT	-	Strop_2809	hypothetical protein
	78	205	3222961	-	CGGGCACTGCGGGTGCT	-	Strop_2817	thioesterase
	75	410	3224293	-	GTGGCTCGACTGGTACT	-	Strop_2818	thiazolinyl imide reductase
	78	126	3241571	-	GCGCCACAGGACTTGCT	-	Strop_2825	cytochrome P450
**8**	PKS		3475927-3510099					
**9**	NRPS		4996301-5013814					
	Score	Distance From ORF	N	Strand	Sequence	Gene	Synonym	Protein Name
	80	426	4997797	-	GTGGCCCCGCTCTTCCT	-	Strop_4414	methyltransferase type 12
	84	166	5003350	-	ATGGCCCGCGCGTTGCG	-	Strop_4416	amino acid adenylation domain-containing protein
	79	496	5009133	-	CTGGCCCGGCGGCTGCC	-	Strop_4419	kynurenine 3-monooxygenase

NC_003155.4 Streptomyces avermitilis MA-4680, complete genome

**1**	PKS		113361-118594					
**2**	PKS		486648-567017					
	Score	Distance From ORF	N	Strand	Sequence	Gene	Synonym	Protein Name
	76	157	490476	-	TTGGCAAGAAAGCGGCA	pteF	SAV_409	LuxR family transcriptional regulator
	77	370	567387	-	CTGGCGAGATTTTCGCA	pteA1	SAV_419	modular polyketide synthase
**3**	NRPS		751402-762285					
**4**	NRPS/PKS		991484-1042269					
	Score	Distance From ORF	N	Strand	Sequence	Gene	Synonym	Protein Name
	75	136	992855	-	CGGGTGCGTCTGTTGCT	cyp4	SAV_838	cytochrome P450
	77	39	1002360	-	CTGGCCCGGCGGTTGGC	-	SAV_845	modular polyketide synthase
	75	51	1021253	-	CTGGCACACAAAGAGCG	nrps7-8	SAV_855	non-ribosomal peptide synthetase
	76	223	1022254	-	TTGGCCGAGGACGTGCT	-	SAV_856	thioesterase
	87	293	1024835	+	CTGGCACGGGCCTTCCA	nrps7-10	SAV_859	non-ribosomal peptide synthetase
	81	433	1026118	+	TTGGCGCGGGCGGTGCA	nrps7-11	SAV_860	non-ribosomal peptide synthetase
	79	488	1036207	+	GTGGCAGGCGAACTGCC	-	SAV_867	ABC transporter ATP-binding protein
**5**	PKS		1132045-1212960					
	Score	Distance From ORF	N	Strand	Sequence	Gene	Synonym	Protein Name
	75	76	1168427	+	ATGGCACCGCACCCGCC	aveC	SAV_940	AveC
	75	111	1208710	-	CAGGCCCGCACCCTGCT	aveBV	SAV_949	dTDP-4-keto-6-deoxyhexose 3,5-epimerase
	76	410	1212380	-	ACGGCCCGGATACTGCA	aveBVIII	SAV_952	dTDP-4-keto-6-deoxy-L-hexose 2,3-reductase
	77	281	1211927	+	GGAGCACTGCTGTTGCT	aveG	SAV_953	thioesterase
	75	114	1212094	+	GTGGGACACGGGCTGCC	aveG	SAV_953	thioesterase
**6**	PKS		1549424-1554224					
**7**	PKS		1893266-1913284					
	Score	Distance From ORF	N	Strand	Sequence	Gene	Synonym	Protein Name
	78	263	1910520	-	GCGGTACGCCAGTTGCC	pks2-2	SAV_1551	modular polyketide synthase
	76	443	1912725	-	CTGGCCCTCCTTGTGCG	pks2-3	SAV_1552	non-ribosomal peptide synthetase
	76	354	1912250	+	CTGGTGCGAGGCGTGCT	-	SAV_1553	methyltransferase

**8**	PKS	2773878-2784841						
**9**	PKS	2877941-2894413						
**10**	PKS	2896543-2914291						
	Score	Distance From ORF	N	Strand	Sequence	Gene	Synonym	Protein Name
	81	339	2904983	-	CTGGCACCGCTCACGCT	-	SAV_2378	polyketide oxidase/hydroxylase
	75	296	2906293	+	ACGGCCCGCCCGCTGCT	-	SAV_2381	TetR family transcriptional regulator
**11**	PKS	3477658-3493243						
	Score	Distance From ORF	N	Strand	Sequence	Gene	Synonym	Protein Name
	77	112	3491806	-	CTGGCCCTGTTCCTGCC	-	SAV_2846	hypothetical protein
	75	159	3492602	-	GAGGCACCCCTTCTGCG	-	SAV_2847	hypothetical protein
	77	210	3493453	-	ACGGCCAGCACCTTGCT	-	SAV_2849	hypothetical protein
**12**	PKS	3534525-3634592						
	Score	Distance From ORF	N	Strand	Sequence	Gene	Synonym	Protein Name
	77	17	3630164	-	ATGGCGCGGCTCGCGCA	olmRII	SAV_2901	LuxR family transcriptional regulator
**13**	NRPS	3930306-3937493						
**14**	NRPS	3980213-3994940						
	Score	Distance From ORF	N	Strand	Sequence	Gene	Synonym	Protein Name
	76	11	3985530	-	CTGGAAGGTACCGTGCA	nrps1-1	SAV_3197	non-ribosomal peptide synthetase
	77	263	3989602	-	CGGGCACGGGACTGGCC	nrps1-2	SAV_3198	non-ribosomal peptide synthetase
**15**	NRPS	4494250-4540450						
	Score	Distance From ORF	N	Strand	Sequence	Gene	Synonym	Protein Name
	79	266	4496242	-	CTGGCGCAGATCCTGCC	pptA4	SAV_3637	phosphopantetheinyl transferase
	83	421	4500418	-	CTGGCCCGGGAACTGCT	-	SAV_3641	ornithine carbamoyltransferase
	80	163	4516448	-	CTGGCACGCCGAGGGCA	nrps2-2	SAV_3643	non-ribosomal peptide synthetase
	76	214	4521073	-	GCGGCACAGCTGTTCCC	cysK2	SAV_3648	cysteine synthase
	77	416	4521275	-	CTGGCGCGCAGCTCGCC	cysK2	SAV_3648	cysteine synthase
	76	211	4523419	-	CTGGCCAGGCTGCTGCG	fadE25	SAV_3650	acyl-CoA dehydrogenase
	75	154	4528046	-	GTGGCCAAGGAGATGCT	-	SAV_3652	isomerase
	75	388	4536085	-	ATGGCGCTGCGGGTGCG	-	SAV_3661	hypothetical protein
**16**	PKS	8553602-8561604						
	Score	Distance From ORF	N	Strand	Sequence	Gene	Synonym	Protein Name
	75	252	8560553	-	CTGGCCCGGACCGAGCT	-	SAV_7185	UDP-glucose:sterol glucosyltransferase
**17**	PKS	8776963-8789766						

NC_003888.3 Streptomyces coelicolor A3(2), complete genome

**1**	PKS	104989-119654

**2**	NRPS	513989-526783
**3**	PKS	1335793-1343779
**4**	NRPS	3543335-3585724
**5**	PKS	5508078-5508078
**6**	PKS	6432566-6465258
**7**	PKS	6895193-6961810
**8**	NRPS	7106284-7116513
**9**	PKS	7590412-7602007
**10**	NRPS	8506283-8523749

NC_010572.1 Streptomyces griseus subsp. griseus NBRC 13350, complete genome

**1**	PKS	294490-307047						
**2**	NRPS	480685-538065						
	Score	Distance From ORF	N	Strand	Sequence	Gene	Synonym	Protein Name
	80	142	535949	-	CTGGCCCCGGTGCTGCT	-	SGR_453	iron ABC transporter
	75	367	536174	-	CTGGCCCGGCCGCTGAA	-	SGR_453	iron ABC transporter
**3**	NRPS	659688-691515						
	Score	Distance From ORF	N	Strand	Sequence	Gene	Synonym	Protein Name
	80	48	664691	+	GTGGCGTGCCTTCTGCA	-	SGR_578	putative SyrP-like protein
	76	167	676251	+	CTGGCCCGGCTGGAGCT	-	SGR_583	putative NRPS
	82	298	685576	-	CTGGCCCGCGCTCTGCT	-	SGR_588	hypothetical protein
**4**	PKS	704021-717508						
	Score	Distance From ORF	N	Strand	Sequence	Gene	Synonym	Protein Name
	76	480	703541	+	GTGCGACGGCGGTTGCC	tebC	SGR_604	putative enediyne biosynthesis protein
	76	474	704168	+	AGGGCACCCGAGGTGCT	unbL1	SGR_605	putative enediyne biosynthesis protein
	79	329	708297	+	GTGGCACGCCAGTGGGA	unbU	SGR_608	putative enediyne biosynthesis protein
**5**	NRPS	762595-774309						
**6**	PKS	937762-968455						
	Score	Distance From ORF	N	Strand	Sequence	Gene	Synonym	Protein Name
	78	299	940053	+	CTGCCACAGCCGATGCA	-	SGR_803	putative large multi-functional protein
	81	130	956182	-	CTGGCACGGCAGCTCCA	-	SGR_813	putative FAD-dependent oxidoreductase
**7**	NRPS	1044652-1071469						
	Score	Distance From ORF	N	Strand	Sequence	Gene	Synonym	Protein Name
	75	294	1046802	-	GGGGCACGCACATCGCC	-	SGR_890	hypothetical protein
	75	437	1060513	-	CTGGCTCACCGGATGCG	-	SGR_896	putative O-methyltransferase
**8**	NRPS/PKS	2924650-2941895						

	Score	Distance From ORF	N	Strand	Sequence	Gene	Synonym	Protein Name
	75	374	2930023	+	ATGGTGCGGCAGTTGGA	-	SGR_2485	putative NRPS
	75	470	2935806	+	ATGGCCCATGTGCTGCC	-	SGR_2486	putative aminopeptidase 2
	78	400	2937233	+	ATGGCGCACGCGGTGCA	-	SGR_2487	putative thioesterase
**9**	NRPS	3048501-3074603						
**10**	NRPS	3762124-3830096						
**11**	PKS	7102079-7171428						
	Score	Distance From ORF	N	Strand	Sequence	Gene	Synonym	Protein Name
	76	362	7161653	+	TCGGCACGCTCGGTGCC	pks1-7	SGR_6083	putative type-I PKS
**12**	PKS	7286041-7354154						
**13**	PKS	7551740-7619551						
	Score	Distance From ORF	N	Strand	Sequence	Gene	Synonym	Protein Name
	83	426	7551314	+	CTGGCAGGGCAGTTGGA	-	SGR_6359	hypothetical protein
	78	256	7570469	-	CTGGGACTGTCCTCGCT	-	SGR_6360	hypothetical protein
	75	422	7574873	-	GTGGCCGACGTAGTGCA	-	SGR_6365	hypothetical protein
	76	266	7596949	-	CTGGCCGGGCACCTGCG	pks3-3	SGR_6371	putative type-I PKS
	81	244	7619795	-	GTGGCCCAGCGGCTGCA	pks3-1	SGR_6373	putative type-I PKS
**14**	NRPS	8011976-8029693						
	Score	Distance From ORF	N	Strand	Sequence	Gene	Synonym	Protein Name
	81	164	8012818	+	GCGGCACGATCCGTGCA	-	SGR_6714	putative ABC-type Fe3+-siderophore transporter substrate-binding protein
	85	326	8015602	+	CTGGCCCGGCTGGTGCT	-	SGR_6716	putative NRPS
	77	424	8030117	-	CTGGCACATCGGCTGGT	-	SGR_6718	putative esterase
**15**	NRPS	8049140-8065069						
**16**	PKS	8100042-8154770						
	Score	Distance From ORF	N	Strand	Sequence	Gene	Synonym	Protein Name
	78	30	8100012	+	CGGGCAGAGTCCTTGCG	pks4-1	SGR_6776	putative NRPS-type-I PKS fusion protein
	77	383	8110593	+	TTCGCACGGACGGTGCG	pks4-2	SGR_6777	putative type-I PKS
	75	269	8124572	+	TTCGGACGCATCGTGCT	-	SGR_6780	putative malonyl-CoA:ACP transacylase

NC_003228.3 Bacteroides fragilis NCTC 9343, complete genome

	Score	Distance From ORF	N	Strand	Sequence	Gene	Synonym	Protein Name
**1**	NRPS	3294535-3306572						
**2**	NRPS	3635663-3639879						
	Score	Distance From ORF	N	Strand	Sequence	Gene	Synonym	Protein Name
	75	357	3638482	+	GCGGCCGACAGCTTGCT	-	BF3118	hypothetical protein

NC_005125.1 Gloeobacter violaceus PCC 7421, complete genome

	Score	Distance From ORF	N	Strand	Sequence	Gene	Synonym	Protein Name
**1**	PKS	2057662-2100787						
	Score	Distance From ORF	N	Strand	Sequence	Gene	Synonym	Protein Name
	76	304	2064191	-	ATGGTCCGCATCTTCCT	-	gll1939	cyclopropane-fatty-acyl-phospholipid synthase

Score	Distance From ORF	N	Strand	Sequence	Gene	Synonym	Protein Name
83	457	2064344	-	CTGGCCCGAGATCTGCT	-	gll1939	cyclopropane-fatty-acyl-phospholipid synthase
77	280	2065156	-	ATGGCCCAGTTGTGGCC	-	gll1940	fatty acid desaturase
77	423	2072927	-	GCGGCCCTGGGGTTGCT	-	gll1946	fatty acid desaturase
81	329	2073836	-	GTGGGGCGGCTTTTGCC	-	gll1947	fatty acid desaturase
75	166	2078085	-	CCGGCAGCACTCCTGCA	-	gll1950	long-chain fatty-acid-CoA ligase
76	219	2078138	-	ATGGCTTCGCACTCGCT	-	gll1950	long-chain fatty-acid-CoA ligase
77	466	2078385	-	GGGGCGCACCCCTTGCG	-	gll1950	long-chain fatty-acid-CoA ligase
81	411	2079094	-	CTGGAGCGCTGTTTGCA	-	gll1951	phosphopantetheinyltransferase family protein
76	461	2088597	-	GTGGCTGGACAGTTGAT	-	gll1954	polyketide synthase
76	146	2088597	-	GTGGCTGGACAGTTGAT	-	gll1955	polyketide synthase
75	317	2088768	-	ATGGCCCTGATCTTTCG	-	gll1955	polyketide synthase
75	277	2094691	-	CTGGACCAGTTGCTGCA	-	gll1957	glycolipid synthase

2 PKS 2999221-3029646

Score	Distance From ORF	N	Strand	Sequence	Gene	Synonym	Protein Name
77	396	2998825	+	CTGGCACCAGAAACGCT	-	glr2821	WD repeat-containing protein
81	288	3009343	-	CTGGCGGGGCCTTTGCG	-	gll2826	hypothetical protein
77	307	3010641	-	ACGGGACATGATTTGCT	-	gll2827	hypothetical protein
80	157	3014822	-	CAGGCGCTGATCTTGCT	-	gll2829	polyketide synthase
79	450	3023483	-	GTGGCACAGACCGTCCT	-	gll2836	alcohol dehydrogenase
79	174	3023483	-	GTGGCACAGACCGTCCT	-	gsl2837	hypothetical protein

3 PKS 3033832-3050434

Score	Distance From ORF	N	Strand	Sequence	Gene	Synonym	Protein Name
75	221	3040466	+	CTGGCGCAGTGTTTGAG	-	glr2850	thioesterase
79	41	3040646	+	CTGGCAACTCTGGTGCA	-	glr2850	thioesterase
78	393	3048049	+	GTGGCCCGTCCGATGCC	-	glr2859	oxidoreductase

4 PKS 4421532-4440489

Score	Distance From ORF	N	Strand	Sequence	Gene	Synonym	Protein Name
75	215	4422391	-	CTGGAAAGTCCATCGCT	-	gll4219	hypothetical protein
83	331	4422933	-	CTGGCCGGAGGATTGCT	-	gll4220	hypothetical protein
75	154	4431563	-	CTGGTAGGCCCCATGCG	-	gll4225	glycolipid synthase

NC_010296.1 Microcystis aeruginosa NIES-843, complete genome

1 NRPS/PKS 2507861-2539146

Score	Distance From ORF	N	Strand	Sequence	Gene	Synonym	Protein Name
77	38	2518358	+	TGGGCGCAAGCCTTGCG	-	MAE_27820	amino acid adenylation
78	118	2526583	+	TGGGCGCGAGCATTGCG	-	MAE_27860	short-chain dehydrogenase/reductase
79	235	2537743	+	GTGGCATCGGTTGTGCG	-	MAE_27910	hypothetical protein

2 NRPS/PKS 3486436-3541027

Score	Distance From ORF	N	Strand	Sequence	Gene	Synonym	Protein Name
79	340	3528001	+	GTGGCAATTGTCCTGCA	mcyF	MAE_38620	McyF protein
79	114	3540068	+	ATGGCAATATTTCTGCA	mcyJ	MAE_38660	McyJ protein

3 NRPS 5194435-5220124

Score	Distance From ORF	N	Strand	Sequence	Gene	Synonym	Protein Name
75	130	5208380	-	TTGGCATGGTATTCTCA	-	MAE_56550	hypothetical protein

	Score	Distance From ORF	N	Strand	Sequence	Gene	Synonym	Protein Name
	80	148	5209406	-	TTGGCTCGACCGTTTCT	-	MAE_56560	bacilysin biosynthesis protein BacA-like protein
	80	198	5209456	-	ATGGCTTAGACCTTGCC	-	MAE_56560	bacilysin biosynthesis protein BacA-like protein
4	-843, complete genome 5519975-5552181 Type: NRPS							

NC_010628.1 Nostoc punctiforme PCC 73102, complete genome

1	PKS		56243-74142					
	Score	Distance From ORF	N	Strand	Sequence	Gene	Synonym	Protein Name
	81	379	58235	-	GTGGCAATATAATTGCC	-	Npun_R0038	short-chain dehydrogenase/reductase SDR
	79	405	60343	-	TTCGCAAAACTATTGCT	-	Npun_R0039	polyketide synthase thioester reductase subunit HglB
	76	291	68964	-	ATGGCCCAAAGTTTTCA	-	Npun_R0042	KR
2	PKS		1549256-1571247					
3	PKS		2521301-2548634					
4	NRPS/PKS		2648282-2708401					
	Score	Distance From ORF	N	Strand	Sequence	Gene	Synonym	Protein Name
	75	183	2660116	-	TTGTCATAGACATTGCC	-	Npun_R2175	KR
	79	484	2664987	-	ATGGCAGGTATAATGCC	-	Npun_R2178	WD-40 repeat-containing protein
	76	206	2704134	+	GTGATACAGTAATTGCA	-	Npun_F2186	alcohol dehydrogenase
	81	362	2705124	+	CAGGCATTCCTATTGCT	-	Npun_F2187	pyrroline-5-carboxylate reductase
5	NRPS		3054973-3081156					
	Score	Distance From ORF	N	Strand	Sequence	Gene	Synonym	Protein Name
	76	50	3055694	+	TGGGCAAGGATATAGCA	-	Npun_F2460	amino acid adenylation domain-containing protein
	76	93	3065615	+	GTGACTCAAGAGTTGCT	-	Npun_F2462	amino acid adenylation domain-containing protein
6	NRPS/PKS		3722118-3780010					
7	NRPS/PKS		3899401-3958883					
	Score	Distance From ORF	N	Strand	Sequence	Gene	Synonym	Protein Name
	75	191	3899210	+	TTCACACGTATGTTGCT	-	Npun_F3155	hypothetical protein
	86	415	3925855	+	ATGGCGTGAATTTTGCT	-	Npun_F3168	hypothetical protein
	76	296	3950844	+	GTGGCTAGGGTGCTGCG	-	Npun_F3173	amino acid adenylation domain-containing protein
	75	148	3950992	+	AAGGCCCTAGTATTGCC	-	Npun_F3173	amino acid adenylation domain-containing protein
	75	54	3955358	+	TAGGGAAGATAATTGCT	-	Npun_F3174	isoprenylcysteine carboxyl methyltransferase
8	PKS		4180429-4210477					
	Score	Distance From ORF	N	Strand	Sequence	Gene	Synonym	Protein Name
	75	482	4181756	-	CTCGAACATTTATTGCT	-	Npun_R3355	thioesterase
	75	474	4185955	+	TTGTCAGGAGGGTTGCC	-	Npun_F3359	beta-ketoacyl synthase
	76	364	4186065	+	CTGGCTAGAGAAATGCT	-	Npun_F3359	beta-ketoacyl synthase

Score	Distance From ORF	N	Strand	Sequence	Gene	Synonym	Protein Name
83	221	4201954	+	ATGGCAGTGATATTGCC	-	Npun_F3363	short-chain dehydrogenase/reductase SDR, HetN

9 NRPS/PKS 4256267-4330313

Score	Distance From ORF	N	Strand	Sequence	Gene	Synonym	Protein Name
81	468	4259675	-	ATGGCAAGAGTTTTGTA	-	Npun_R3419	thioesterase
81	165	4259675	-	ATGGCAAGAGTTTTGTA	-	Npun_R3420	MbtH domain-containing protein
76	491	4260403	-	ATTGCACGAGGCTTTCT	-	Npun_R3421	hypothetical protein
77	499	4280094	-	ACGGCCCGACTGTAGCT	-	Npun_R3429	condensation domain-containing protein
76	482	4286311	-	TTGGATCGGCGATCGCT	-	Npun_R3430	beta-ketoacyl synthase
75	239	4307280	-	ATGGCTCTCTTATTGAA	-	Npun_R3436	amino acid adenylation domain-containing protein
78	331	4307372	-	TTGGCATCCAGCTTGTT	-	Npun_R3436	amino acid adenylation domain-containing protein
81	358	4321256	-	ATGGCAGCACTTGTGCA	-	Npun_R3448	taurine catabolism dioxygenase TauD/TfdA

10 NRPS/PKS 8150738-8167006

NC_008536.1 Solibacter usitatus Ellin6076, complete genome

1 PKS 2480784-2494608

Score	Distance From ORF	N	Strand	Sequence	Gene	Synonym	Protein Name
80	22	2489341	+	CTGGCCGGGAGATTGCG	-	Acid_1973	beta-ketoacyl synthase
77	463	2493837	+	ACGGCAGACCCATTGCC	-	Acid_1974	putative acyl carrier protein

2 PKS 3884932-3895290

Score	Distance From ORF	N	Strand	Sequence	Gene	Synonym	Protein Name
75	213	3885870	-	GTGGATCGAGTTTCGCT	-	Acid_3076	4'-phosphopantetheinyl transferase
78	300	3885957	-	ATGGCACCGCGGCTGAT	-	Acid_3076	4'-phosphopantetheinyl transferase
77	50	3893616	-	CGGGCACGCCGCTTGAG	-	Acid_3077	beta-ketoacyl synthase

NC_009674.1 Bacillus cereus subsp. cytotoxis NVH 391-98, complete genome

1 NRPS 417602-429629

Score	Distance From ORF	N	Strand	Sequence	Gene	Synonym	Protein Name
80	491	422474	+	CTGGTAGAGGATTTGCA	-	Bcer98_0368	amino acid adenylation domain-containing protein

2 NRPS 1197549-1205485

Score	Distance From ORF	N	Strand	Sequence	Gene	Synonym	Protein Name
77	484	1198103	+	ACGTCACGAGGTTTGCA	-	Bcer98_1088	2-nitropropane dioxygenase NPD

3 NRPS 1853248-1878954

Score	Distance From ORF	N	Strand	Sequence	Gene	Synonym	Protein Name
76	310	1855588	+	TTGGAGCGTCTTTGGCT	-	Bcer98_1744	AMP-dependent synthetase and ligase
80	293	1856961	+	TAGGCGCTGGTGTTGCT	-	Bcer98_1745	hypothetical protein

4 PKS 3245067-3256221

**5**	PKS	1781906-1859783						
	Score	Distance From ORF	N	Strand	Sequence	Gene	Synonym	Protein Name
	77	130	1785974	+	CCGGCACCTTAGCTGCT	pksE	BSU17120	enzyme involved in polyketide synthesis
	81	446	1791566	+	CTGCCACAGATATTGCC	pksI	BSU17170	polyketide biosynthesis enoyl-CoA hydratase
**6**	NRPS	1949682-2002351						
	Score	Distance From ORF	N	Strand	Sequence	Gene	Synonym	Protein Name
	81	138	1956312	-	GTGGAACGGATGCTGCG	yngI	BSU18250	AMP-binding domain protein
	82	303	1956477	-	CTGGCACGGCAAATGGT	yngI	BSU18250	AMP-binding domain protein
	79	340	1957700	-	CGGGCACTGCTCTTGTT	yngJ	BSU18260	acyl-CoA dehydrogenase, short-chain specific
	76	263	1960350	-	ATGGGAAGGCGCCTGCG	yngL	BSU18290	putative integral inner membrane protein
	77	161	1975017	-	ATTGAACACTTTTTGCT	ppsD	BSU18310	plipastatin synthetase
**7**	NRPS	3280519-3297919						
	Score	Distance From ORF	N	Strand	Sequence	Gene	Synonym	Protein Name
	75	391	3290651	-	GTGGCACCTGTCCAGCG	dhbE	BSU31980	2,3-dihydroxybenzoate-AMP ligase
	82	299	3291784	-	TTGGCCTTGAGCTTGCA	dhbC	BSU31990	isochorismate synthase DhbC
	78	267	3296537	-	CAGGCACAGCGACTGCC	yuiF	BSU32040	amino acid transporter
	80	328	3296598	-	ATGGCAGGAACGTTTCT	yuiF	BSU32040	amino acid transporter
**8**	NRPS	3952275-3958482						
	Score	Distance From ORF	N	Strand	Sequence	Gene	Synonym	Protein Name
	78	347	3957044	+	ATGGCACCGCCTATGGT	ywaA	BSU38550	branched-chain amino acid aminotransferase

NC_003030.1 Clostridium acetobutylicum ATCC 824, complete genome

**1**	PKS	3529432-3534819

NC_009706.1 Clostridium kluyveri DSM 555, complete genome

**1**	NRPS	1554646-1602560						
	Score	Distance From ORF	N	Strand	Sequence	Gene	Synonym	Protein Name
	75	321	1564208	+	CTCGAATGGATATTGCT	-	CKL_1507	NRPS cyclization domain containing protein
	77	233	1578681	+	GAGGCATAGAACATGCA	-	CKL_1515	hypothetical protein
**2**	NRPS/PKS	1818097-1833491						
**3**	NRPS/PKS	2414655-2426709						
	Score	Distance From ORF	N	Strand	Sequence	Gene	Synonym	Protein Name
	76	361	2419626	-	TTGATACAGTTTTTGCA	-	CKL_2354	nonribosomal peptide synthetase

NC_008497.1 Lactobacillus brevis ATCC 367, complete genome

**1**	NRPS	1293616-1300116						
	Score	Distance From ORF	N	Strand	Sequence	Gene	Synonym	Protein Name
	75	122	1297040	-	GTGCAACTGTTGTTGCA	-	LVIS_1323	D-alanyl transfer protein

Score	Distance From ORF	N	Strand	Sequence	Gene	Synonym	Protein Name
81	491	1300607	-	GGGGCACCACGGTTGCG	-	LVIS_1327	D-Ala-teichoic acid biosynthesis protein (putative)

NC_002973.6 Listeria monocytogenes str. 4b F2365, complete genome

**1**	NRPS	1002948-1007380

NC_002952.2 Staphylococcus aureus subsp. aureus MRSA252, complete genome

1 NRPS 198393-206225

Score	Distance From ORF	N	Strand	Sequence	Gene	Synonym	Protein Name
81	487	197906	+	CTAGCATGCTTGTTGCT	-	SAR0180	putative non-ribosomal peptide synthetase

NC_003063.2 Agrobacterium tumefaciens str. C58 chromosome linear, complete sequence

1 NRPS 74687-80557

Score	Distance From ORF	N	Strand	Sequence	Gene	Synonym	Protein Name
78	121	79699	-	ATGGCTGCTCGGTTGCT	-	Atu3073	aspartate racemase
83	322	80879	-	CCGGCACGGCGCTCGCT	-	Atu3074	short chain dehydrogenase

2 NRPS/PKS 716058-769035

Score	Distance From ORF	N	Strand	Sequence	Gene	Synonym	Protein Name
78	463	729910	-	CTGGTCCGGATTTCGCT	-	Atu3673	siderophore biosynthesis protein
78	187	729910	-	CTGGTCCGGATTTCGCT	-	Atu3674	hypothetical protein
85	425	734796	+	ATGGCACGCTACTGGCG	-	Atu3677	polyketide synthase, siderophore biosynthesis protein
75	293	734928	+	GCGGCGCGTTTCTCGCT	-	Atu3677	polyketide synthase, siderophore biosynthesis protein
75	28	737448	+	ATGCAAGGCTTCTTGCA	-	Atu3680	putative siderophore biosynthesis protein
80	62	742445	+	ATGGCAGCGCTGTTGAT	-	Atu3682	non-ribosomal peptide synthetase, siderophore biosynthesis protein
86	328	765024	+	TTGGCACGGATGTCGCC	fecD	Atu3690	ABC transporter, membrane spanning protein (iron (III) dicitrate)
79	247	765105	+	CCGGCAGCGCCGTTGCA	fecD	Atu3690	ABC transporter, membrane spanning protein (iron (III) dicitrate)
75	470	767004	+	CTGGCGCCCCAGGTGCC	-	Atu3692	sigma factor

NC_012988.1 Methylobacterium extorquens DM4, complete genome

**1**	PKS	2613383-2622159

NC_012988.1 Methylobacterium extorquens DM4, complete genome

**1**	NRPS	4859328-4866323

rquens DM4, complete genome 5649466-5678659 Type: NRPS

Score	Distance From ORF	N	Strand	Sequence	Gene	Synonym	Protein Name

86	134	5649332	+	CAGGCACAGGCTTTGCT	-	METDI5670	putative UDP-N-acetyl-D-mannosaminuronic acid dehydrogenase
77	53	5653547	+	ATGGCGCCCGTCTTCCT	-	METDI5674	putative glycosyl transferase
75	105	5662917	-	CCGGCAACCACCTTGCC	-	METDI5678	hypothetical protein
75	46	5667529	-	ATGGCAGGTCCGCCGCT	-	METDI5683	hypothetical protein
75	148	5675379	-	CTGGCACCGCGCTTCGA	murE	METDI5688	UDP-N-acetylmuramoylalanyl-D-glutamate--2, 6-diaminopimelate ligase
75	355	5677962	-	TTGGTGCGCGGCCTGCA	-	METDI5690	hypothetical protein

NC_003296.1 Ralstonia solanacearum GMI1000 plasmid pGMI1000MP, complete sequence

**1**	NRPS/PKS		782247-820777					
	Score	Distance From ORF	N	Strand	Sequence	Gene	Synonym	Protein Name
	76	194	802722	+	CTGGCCGAACTCTGGCA	RSp0642	RS05859	peptide synthetase protein
**2**	NRPS		1782597-1797233					
	Score	Distance From ORF	N	Strand	Sequence	Gene	Synonym	Protein Name
	76	13	1787443	+	CTCCCACCGCTTTTGCA	RSp1421	RS03121	hypothetical protein
**3**	NRPS/PKS		1945814-1979076					
	Score	Distance From ORF	N	Strand	Sequence	Gene	Synonym	Protein Name
	79	452	1949544	-	GTCGCACGCGATCTGCA	tISRso15	RSp1547	ISRSO15-transposase protein
	83	50	1950406	-	GTGGCACGTTCAATGCG	RSp1549	RS02105	hypothetical protein
	82	103	1951581	-	CCGGCATTGCTTTTGCA	RSp1551	RS02107	hypothetical protein
	80	248	1951726	-	GTGGCGCTGCTGGTGCA	RSp1551	RS02107	hypothetical protein
	75	279	1957715	+	GTGGGATGGGATATGCC	hexR	RSp1556	putative transcription regulation repressor HEXR transcription regulator protein
	75	60	1957934	+	GCGGCCCCGCGCTTGCC	hexR	RSp1556	putative transcription regulation repressor HEXR transcription regulator protein
	77	289	1960873	-	GCGGCACTGGGCCTGCA	pgl	RSp1558	6-phosphogluconolactonase oxidoreductase protein
	77	106	1962311	+	GAGGCACGCAATTCGCG	edd	RSp1560	phosphogluconate dehydratase
	78	135	1964859	-	CGGGCATCGTTGTTGCG	RSp1561	RS02117	hypothetical protein
	80	247	1964633	+	ATGGCGCGGATGGTGCC	RSp1562	RS02118	hypothetical protein
	77	139	1965305	+	TGCGCATGGATTTTGCA	RSp1563	RS02119	hypothetical protein
	75	312	1974525	-	CTGGAATGGCGTTAGCG	RSp1571	RS02127	transcriptional regulator transcription regulator protein

NC_008095.1 Myxococcus xanthus DK 1622, complete genome

**1**	NRPS		1488859-1520119					
	Score	Distance From ORF	N	Strand	Sequence	Gene	Synonym	Protein Name
	82	476	1490324	+	GTGGCGCGGCCCTTGAA	-	MXAN_1276	glutamate-cysteine ligase family 2 protein
	77	461	1497379	-	TCTGGAACGCGTGCAGCAGGGG	-	MXAN_1281	hypothetical protein
	75	1	1500305	+	AGGGCAAGGCATTTCCA	-	MXAN_1284	2-isopropylmalate synthase/homocitrate synthase family protein

	Score	Distance From ORF	N	Strand	Sequence	Gene	Synonym	Protein Name
	75	159	1503794	+	TTGGACCGGCTCGTGCG	abcA	MXAN_1286	ABC transporter permease/ATP-binding protein
	75	63	1503890	+	TTGGCGCGGAAGGCGCA	abcA	MXAN_1286	ABC transporter permease/ATP-binding protein
	76	304	1505775	+	CATTGCCCGCGCCCTGCTGCTG	-	MXAN_1287	hypothetical protein
	78	233	1505846	+	GAGGCCCAACTGTTGCG	-	MXAN_1287	hypothetical protein
	77	252	1517769	-	CCCTGGCACGTGACGTGTTCAA	-	MXAN_1291	non-ribosomal peptide synthetase
	79	252	1517769	-	CCCTGGCACGTGACGTGTTCA	-	MXAN_1291	non-ribosomal peptide synthetase
	80	250	1517767	-	CTGGCACGTGACGTGTT	-	MXAN_1291	non-ribosomal peptide synthetase
	76	460	1517977	-	GTGGTGCGGCGGATGCA	-	MXAN_1291	non-ribosomal peptide synthetase
	76	455	1518967	-	GCTGGCGCGCGAGGAACTGAAG	-	MXAN_1292	hypothetical protein
	75	300	1520419	-	ACCGGTACACACGTTGGTGGCG	hemG	MXAN_1293	protoporphyrinogen oxidase
	76	336	1520455	-	GGAGACACCGGCGTTGTTGATG	hemG	MXAN_1293	protoporphyrinogen oxidase
**2**	NRPS		1832662-1910318					
	Score	Distance From ORF	N	Strand	Sequence	Gene	Synonym	Protein Name
	75	298	1834249	-	GTGGCACGTCGCTGGTG	-	MXAN_1560	class I aminotransferase
	79	84	1836766	-	TCTGGCACGGTGACTGCTAAGC	ahpC	MXAN_1564	alkyl hydroperoxide reductase C
	89	85	1836767	-	TTCTGGCACGGTGACTGCTAAG	ahpC	MXAN_1564	alkyl hydroperoxide reductase C
	89	83	1836765	-	CTGGCACGGTGACTGCT	ahpC	MXAN_1564	alkyl hydroperoxide reductase C
	88	85	1836767	-	TTCTGGCACGGTGACTGCTAA	ahpC	MXAN_1564	alkyl hydroperoxide reductase C
	77	420	1841516	-	CGCTGGCGCGGTTGCTGCGCAT	-	MXAN_1567	urea amidolyase-like protein
	79	420	1841516	-	CGCTGGCGCGGTTGCTGCGCA	-	MXAN_1567	urea amidolyase-like protein
	80	418	1841514	-	CTGGCGCGGTTGCTGCG	-	MXAN_1567	urea amidolyase-like protein
	76	61	1842452	-	GGCTGGCACGCCAGCGTCTCCG	-	MXAN_1568	LamB/YcsF family/allophanate hydrolase family protein
	78	61	1842452	-	GGCTGGCACGCCAGCGTCTCC	-	MXAN_1568	LamB/YcsF family/allophanate hydrolase family protein
	75	461	1842852	-	AGCTGCCACGCGCCTGGCGCG	-	MXAN_1568	LamB/YcsF family/allophanate hydrolase family protein
	76	293	1844083	+	ATGACTCGCAGCTTGCG	-	MXAN_1570	class V aminotransferase
	75	376	1847137	+	GCTGGCCTCCACGGTGGTGACG	-	MXAN_1573	AMP-binding domain protein
	77	227	1849786	-	CCTGGCGCGCGCCGGGGTGAAG	-	MXAN_1574	TfoX domain-containing protein
	75	60	1854462	-	CACGGCGCAGCGCTTGCTAAGG	-	MXAN_1578	metallo-beta-lactamase family protein
	75	61	1854463	-	CCACGGCGCAGCGCTTGCTAAG	-	MXAN_1578	metallo-beta-lactamase family protein
	81	59	1854461	-	ACGGCGCAGCGCTTGCT	-	MXAN_1578	metallo-beta-lactamase family protein
	76	104	1854518	+	GGGGCGCAATCCTTGCG	-	MXAN_1579	hypothetical protein
	77	75	1854547	+	GGGGCGCAGGTCTTGCG	-	MXAN_1579	hypothetical protein
	80	180	1860605	-	AAGTGGCGCGGCCGCTGCTTGG	-	MXAN_1584	hypothetical protein
	80	180	1860605	-	AAGTGGCGCGGCCGCTGCTTG	-	MXAN_1584	hypothetical protein
	84	178	1860603	-	GTGGCGCGGCCGCTGCT	-	MXAN_1584	hypothetical protein
	75	146	1861734	+	GTGGGACATCCGGTGCG	-	MXAN_1587	hypothetical protein
	76	130	1866438	-	GGAGGCACGCCGCACGCTGGAA	-	MXAN_1590	putative para-aminobenzoate synthase, component I
	75	190	1871142	-	CCTGCTCTGCACGGAGCTGAAG	-	MXAN_1592	hypothetical protein

	75	203	1871155	-	GGCTGGAAGTGGACCTGCTCTG	-	MXAN_1592	hypothetical protein
	77	203	1871155	-	GGCTGGAAGTGGACCTGCTCT	-	MXAN_1592	hypothetical protein
	78	130	1875006	-	CATGGCACGGGGGACGAAGAGG	-	MXAN_1595	hypothetical protein
	75	428	1875304	-	GGAGGATGACCGGCTGCTGATG	-	MXAN_1595	hypothetical protein
	76	277	1877522	+	GGTGGCCAGCCGGGCGCGGACG	-	MXAN_1599	putative methyltransferase
	75	44	1877755	+	GTGGTGCCGCTGCTGCT	-	MXAN_1599	putative methyltransferase
	75	398	1878919	+	GGAGGCCCGCTCCGAGCTGTGG	-	MXAN_1600	class I aminotransferase
	79	267	1879050	+	GGCTGGCTCGGGCGTTGCGTCC	-	MXAN_1600	class I aminotransferase
	85	270	1879047	+	CTGGCTCGGGCGTTGCG	-	MXAN_1600	class I aminotransferase
	80	268	1879049	+	GGCTGGCTCGGGCGTTGCGTC	-	MXAN_1600	class I aminotransferase
	79	398	1881868	-	GTGGCCCGGGCACTGCG	-	MXAN_1601	fatty acid desaturase family protein
	76	297	1883277	+	GTGGCCTGGGTGGTGCC	-	MXAN_1603	putative non-ribosomal peptide synthetase
	78	262	1889174	+	TGCTGGAAGGGTCGCTGCTGGG	-	MXAN_1605	putative permease
	79	263	1889173	+	TGCTGGAAGGGTCGCTGCTGG	-	MXAN_1605	putative permease
	78	265	1889171	+	CTGGAAGGGTCGCTGCT	-	MXAN_1605	putative permease
	75	256	1890469	+	GGTGGCCGCCTTCATGCTGGAG	-	MXAN_1606	hypothetical protein
	75	260	1890465	+	GTGGCCGCCTTCATGCT	-	MXAN_1606	hypothetical protein
	75	294	1910612	-	GTAGGAACGCACGACCCTCAAG	-	MXAN_1608	hypothetical protein
**3**	NRPS/PKS		3251570-3266656					
**4**	PKS		4029025-4040172					
	Score	Distance From ORF	N	Strand	Sequence	Gene	Synonym	Protein Name
	76	178	4029404	+	GCTGGTCCACATCATGCTCGAG	-	MXAN_3460	Gfo/Idh/MocA family oxidoreductase
	76	182	4029400	+	CTGGTCCACATCATGCT	-	MXAN_3460	Gfo/Idh/MocA family oxidoreductase
**5**	NRPS/PKS		4220394-4350875					
	Score	Distance From ORF	N	Strand	Sequence	Gene	Synonym	Protein Name
	75	319	4254532	-	ATGGCGGAAGAGCTGCT	-	MXAN_3627	hypothetical protein
	75	398	4255390	-	ACTGCCACGCCAGCAGCTCGGG	-	MXAN_3628	non-ribosomal peptide biosynthesis thioesterase
	75	399	4255391	-	AACTGCCACGCCAGCAGCTCG	-	MXAN_3628	non-ribosomal peptide biosynthesis thioesterase
	75	308	4259689	-	CTGGCGGACATCTGGCA	-	MXAN_3630	polyketide synthase type I
	75	287	4264309	-	GAAGGAACAGCTGTTGCGCATG	-	MXAN_3631	polyketide synthase type I
	78	286	4264308	-	AAGGAACAGCTGTTGCG	-	MXAN_3631	polyketide synthase type I
	76	211	4284118	+	AAGGCGTGCGCATTGCT	-	MXAN_3635	non-ribosomal peptide synthase/polyketide synthase
	77	32	4334952	+	CTGGTACTTCGAGTGCA	-	MXAN_3638	M19 family peptidase
	76	311	4347401	-	GTGGAAAACCATCTGCT	-	MXAN_3644	isochorismatase
	75	268	4351143	-	GTGGCACGCGAAATCCC	-	MXAN_3647	2,3-dihydroxybenzoate-2,3-dehydrogenase
**6**	PKS		4498820-4544105					
	Score	Distance From ORF	N	Strand	Sequence	Gene	Synonym	Protein Name
	76	284	4498536	+	TTGAGACGGAGATTGCG	-	MXAN_3778	DnaK family protein
**7**	PKS		4729983-4809194					
	Score	Distance From ORF	N	Strand	Sequence	Gene	Synonym	Protein Name
	75	337	4731192	-	CGTGGGCCGGTTCCGGCTCAAG	-	MXAN_3931	hypothetical protein
	76	73	4740097	-	TCAGACACGCCCATCGCTGATC	-	MXAN_3932	polyketide synthase
	75	90	4740114	-	CTTGAAAAACCGGTTGCTCAGA	-	MXAN_3932	polyketide synthase
	75	103	4755572	-	AGGTGGTACTTCGCTTACTCG	-	MXAN_3933	mixed type I polyketide synthase - peptide synthetase

	Score	Distance From ORF	N	Strand	Sequence	Gene	Synonym	Protein Name
	76	101	4755570	-	GTGGTACTTCGCTTACT	-	MXAN_3933	mixed type I polyketide synthase - peptide synthetase
	75	428	4784222	-	ATTGGCTCGCGTCTGGCTGCGC	-	MXAN_3935	non-ribosomal peptide synthase/polyketide synthase Ta1
	77	221	4784015	-	TGCTGGCACGAACCCTGGGCG	-	MXAN_3935	non-ribosomal peptide synthase/polyketide synthase Ta1
	82	427	4784221	-	TTGGCTCGCGTCTGGCT	-	MXAN_3935	non-ribosomal peptide synthase/polyketide synthase Ta1
	78	219	4784013	-	CTGGCACGAACCCTGGG	-	MXAN_3935	non-ribosomal peptide synthase/polyketide synthase Ta1
	75	315	4784109	-	TTGGCGCTGAGCGTGCC	-	MXAN_3935	non-ribosomal peptide synthase/polyketide synthase Ta1
	78	333	4790662	-	GTTGGTCCGCCGGGCGGTGGAG	-	MXAN_3936	polyketide synthase
	75	155	4797857	-	GCTGTCGCTCGTGACGCTCAAG	-	MXAN_3938	polyketide synthase
	76	245	4800766	-	GGCTGGCTCTGCAACTGCGCAG	-	MXAN_3941	polyketide beta-ketoacyl:acyl carrier protein synthase
	76	245	4800766	-	GGCTGGCTCTGCAACTGCGCA	-	MXAN_3941	polyketide beta-ketoacyl:acyl carrier protein synthase
	75	243	4800764	-	CTGGCTCTGCAACTGCG	-	MXAN_3941	polyketide beta-ketoacyl:acyl carrier protein synthase
	77	318	4804366	-	GTGGCGCATGCCTTTCT	-	MXAN_3943	cytochrome P450 family protein
	77	97	4805951	-	CCTGGACAGCCTGCGGCTGCAG	taF	MXAN_3945	polyketide TA biosynthesis protein TaF
	76	464	4806318	-	CCTGTCGCGCGTGGTGCTGGAC	taF	MXAN_3945	polyketide TA biosynthesis protein TaF
	76	220	4806318	-	CCTGTCGCGCGTGGTGCTGGAC	-	MXAN_3946	putative acyl carier protein
**8**	NRPS/PKS		4875470-4906976					
	Score	Distance From ORF	N	Strand	Sequence	Gene	Synonym	Protein Name
	79	135	4900530	-	CTCGCCCGCAGGTTGCA	-	MXAN_4001	non-ribosomal peptide synthase/polyketide synthase
	75	470	4906209	-	AGGGCACGGCCCGTGAT	-	MXAN_4002	nonribosomal peptide synthetase
**9**	NRPS/PKS		4996865-5026357					
**10**	NRPS/PKS		5252320-5298262					
	Score	Distance From ORF	N	Strand	Sequence	Gene	Synonym	Protein Name
	76	350	5253698	+	GCTGGAGCGCGTGCTGCTGGCC	-	MXAN_4290	putative thioesterase
	77	352	5253696	+	TGCTGGAGCGCGTGCTGCTGG	-	MXAN_4290	putative thioesterase
	76	354	5253694	+	CTGGAGCGCGTGCTGCT	-	MXAN_4290	putative thioesterase
	76	132	5263568	-	ATGTCGCCGTGCTTGCA	-	MXAN_4295	patatin-like phospholipase family protein
	77	150	5267893	-	AGCTGGGAGTGGACCTGCTCA	-	MXAN_4296	non-ribosomal peptide synthetase
	77	379	5279177	-	GATGCGTCGGCAGTTGCTGATG	-	MXAN_4298	polyketide synthase type I
	80	178	5287908	-	ATGGCGCTCGAGTTGCG	-	MXAN_4299	non-ribosomal peptide synthase/polyketide synthase
	77	183	5292178	-	CGCTGGCGAAGCGGCTGCTGT	-	MXAN_4300	polyketide synthase type I
	76	181	5292176	-	CTGGCGAAGCGGCTGCT	-	MXAN_4300	polyketide synthase type I
**11**	PKS		5407416-5440677					
**12**	NRPS/PKS		5444807-5471033					

	Score	Distance From ORF	N	Strand	Sequence	Gene	Synonym	Protein Name
	75	182	5448069	+	CCTGGACGACCGGTTGGTGCTG	-	MXAN_4413	hypothetical protein
	78	328	5468596	+	CCTGGGCCACGGATTGCTGCGC	-	MXAN_4416	cephalosporin hydroxylase family protein
	79	332	5468592	+	CTGGGCCACGGATTGCT	-	MXAN_4416	cephalosporin hydroxylase family protein
**13**	NRPS/PKS	5600883-5653548						
	Score	Distance From ORF	N	Strand	Sequence	Gene	Synonym	Protein Name
	75	259	5612126	-	ATGGCGCTGGAGCTGCG	-	MXAN_4526	polyketide synthase type I
	78	373	5612240	-	ATGGCGCTGCTGTTGGA	-	MXAN_4526	polyketide synthase type I
	81	466	5612333	-	GTGGCAGGGCAGGTGCG	-	MXAN_4526	polyketide synthase type I
	75	170	5627582	-	CTGGCTGAACAACTGCA	-	MXAN_4527	polyketide synthase
	79	187	5653735	-	GTGGCACAAGCTGCGCT	-	MXAN_4532	non-ribosomal peptide synthase
**14**	NRPS	5735538-5780049						
	Score	Distance From ORF	N	Strand	Sequence	Gene	Synonym	Protein Name
	75	39	5755794	+	CAATGGAACGGCGCATCCTCC	-	MXAN_4598	non-ribosomal peptide synthase
	75	41	5755792	+	ATGGAACGGCGCATCCT	-	MXAN_4598	non-ribosomal peptide synthase
	75	485	5759989	+	CCTGGGCTACGGGGTGCTGCGC	-	MXAN_4599	M28 family peptidase
	75	493	5765386	-	GGTGGCCTGGGCGTCGGTGGCG	-	MXAN_4600	radical SAM domain-containing protein
	75	40	5764933	-	GCGGTAAAGTCTTTGCT	-	MXAN_4600	radical SAM domain-containing protein
	78	237	5774788	+	CCTGGCCCGTCTGGAGCTCCAG	-	MXAN_4602	hypothetical protein
	75	241	5774784	+	CTGGCCCGTCTGGAGCT	-	MXAN_4602	hypothetical protein
	76	62	5777754	+	ATGTCGCTCGACTTGCT	-	MXAN_4604	hypothetical protein

NC_010162.1 Sorangium cellulosum 'So ce 56', complete genome

**1**	PKS	488146-499710						
**2**	PKS	1150403-1167578						
	Score	Distance From ORF	N	Strand	Sequence	Gene	Synonym	Protein Name
	76	300	1150103	+	CTGGCCTGCGCGTCGCT	-	sce0818	putative dioxygenase
	75	299	1151780	+	ATGGCGCTGATCTTCCG	-	sce0819	polyketide synthase
**3**	NRPS	3204860-3216562						
**4**	PKS	4376667-4461851						
	Score	Distance From ORF	N	Strand	Sequence	Gene	Synonym	Protein Name
	84	488	4376179	+	CTGGCAGGATATCTGCT	-	sce3188	polyketide synthase
	77	52	4440389	+	CTGGGGCGGATCTCGCT	-	sce3193	polyketide synthase
**5**	PKS	5761328-5853706						
**6**	PKS	6867494-6856887						
**7**	PKS	9402480-9513516						
	Score	Distance From ORF	N	Strand	Sequence	Gene	Synonym	Protein Name
	81	172	9407529	-	TCGGCACGCTGCCTGCA	-	sce6747	hypothetical protein
	90	68	9411437	-	GTGGCGCGCCGCTTGCT	-	sce6751	hypothetical protein
	75	485	9425010	-	GTGGCGCCGCGGCTGCC	-	sce6759	hypothetical protein
	75	4	9436354	+	ATGGCAGGAGGGGTGAT	-	sce6766	putative secreted protein

Score	Distance From ORF	N	Strand	Sequence	Gene	Synonym	Protein Name
81	80	9440680	-	CGGGCACGGGCGCTGCT	-	sce6770	hypothetical protein
80	438	9441038	-	AGGGCATGATCGTTGCC	-	sce6770	hypothetical protein
77	38	9452036	-	GCGGTACACCCCTTGCG	-	sce6780	hypothetical protein
75	93	9452091	-	GAGGCGCGGCGGATGCT	-	sce6780	hypothetical protein
81	344	9452342	-	CTGGCTCGAGGCCTGCA	-	sce6780	hypothetical protein
75	212	9452057	+	GCGGCCCGCCCGCTGCT	-	sce6781	TetR family transcriptional regulator
77	479	9456589	+	ACGGCACGCGACGTGCG	-	sce6785	hypothetical protein
77	260	9460836	+	CTGGCACCGGGCGAGCT	-	sce6787	hypothetical protein
78	361	9466227	-	CTGGCGCTCGATCTGCT	-	sce6792	hypothetical protein
77	107	9470202	-	TTCGCATGGCTGTTGGA	-	sce6797	hypothetical protein
75	293	9479287	-	ATGGTTCGTTTCTCGCT	-	sce6805	hypothetical protein
77	345	9497084	-	GTGGATCGAGTCATGCT	-	sce6819	hypothetical protein

8 NRPS/PKS 11424644-11529102

Score	Distance From ORF	N	Strand	Sequence	Gene	Synonym	Protein Name
81	427	11427264	-	GGGGCACGATCGCTGCT	-	sce8207	sigma-54 dependent transcriptional regulator
84	42	11432421	-	CTGGCATCCCTCGTGCT	-	sce8210	putative DNA helicase
75	419	11456144	+	AGGGCGCGGCGTCTGCA	-	sce8233	hypothetical protein
82	43	11458261	+	AGGGCACGGCTCTTGGA	-	sce8234	hypothetical protein
76	173	11460728	+	GTGGCCGACGTCCTGCT	yfbL2	sce8235	putative aminopeptidases
75	388	11472575	+	GTGGCGCGCTCCCCGCT	-	sce8244	putative protein phosphatase
75	371	11475747	-	GAGGCCCAGCTCGTGCA	-	sce8246	CobW/P47K family protein

NC_002163.1 Campylobacter jejuni subsp. jejuni NCTC 11168, complete genome

1 NRPS 1236809-1238584

NC_000913.2 Escherichia coli str. K-12 substr. MG1655, complete genome

1 NRPS 608682-631222

Score	Distance From ORF	N	Strand	Sequence	Gene	Synonym	Protein Name
78	373	612792	+	ATCGCACCGTGGTTGCC	ybdZ	b4511	conserved protein
80	361	619783	-	CTGGTACGCCGGATGCG	fepC	b0588	iron-enterobactin transporter subunit
79	153	620564	-	CCGTCACGCTACTTGCT	fepG	b0589	iron-enterobactin transporter subunit
79	138	621385	+	CGGGCACGGCAATGGCG	entS	b0591	enterobactin exporter, iron-regulated
76	428	624161	-	TGGGCGCAAGTGTTGCC	fepB	b0592	iron-enterobactin transporter subunit
79	291	625002	+	CTGGCCTGTCTGCTGCA	entE	b0594	2,3-dihydroxybenzoate-AMP ligase component of enterobactin synthase multienzyme complex

NC_002942.5 Legionella pneumophila subsp. pneumophila str. Philadelphia 1, complete genome

1 NRPS/PKS 2166115-2171151

2 NRPS/PKS 2442757-2470485

NC_002516.2 Pseudomonas aeruginosa PAO1, complete genome

1 NRPS 2532669-2549458

Score	Distance From ORF	N	Strand	Sequence	Gene	Synonym	Protein Name
90	276	2539319	-	CTGGCCCGGGCCTTGCT	-	PA2302	non-ribosomal peptide synthetase

Score	Distance From ORF	N	Strand	Sequence	Gene	Synonym	Protein Name
79	154	2545100	-	ATGGCGCTGCTGTTGGT	-	PA2305	non-ribosomal peptide synthetase
83	8	2545667	-	ATGGAACGAATGTCGCT	-	PA2306	hypothetical protein
84	55	2547555	-	CTGGCAGGACTATTTCT	-	PA2308	ABC transporter ATP-binding protein

2 NRPS 2636517-2687178

Score	Distance From ORF	N	Strand	Sequence	Gene	Synonym	Protein Name
75	125	2640910	+	ACGGCACTGTCCTTCCA	fpvR	PA2388	FpvR
80	88	2651219	+	CTGGCGCCGAGCCTGCT	pvdO	PA2395	PvdO
75	140	2653197	-	CTTCCACGTAAATTGCA	pvdF	PA2396	pyoverdine synthetase F
77	421	2665565	-	CTGGCGCGGATGCCGCT	pvdD	PA2399	pyoverdine synthetase D
77	479	2672108	-	CTGGCGCGGATGCCGCT	pvdJ	PA2400	PvdJ

3 NRPS 4724639-4746551

Score	Distance From ORF	N	Strand	Sequence	Gene	Synonym	Protein Name
81	393	4727194	-	CTGGCTCGCGGGCTGCT	fptA	PA4221	Fe(III)-pyochelin outer membrane receptor precursor
78	265	4728882	-	CCGGCGAGCCTGTTGCT	-	PA4222	ABC transporter ATP-binding protein
80	286	4728903	-	CTGGCCCGTGCGCTGCT	-	PA4222	ABC transporter ATP-binding protein
75	43	4731415	-	CTGGCGCAATCCTTGTC	pchG	PA4224	pyochelin biosynthetic protein PchG
75	16	4736814	-	CTGGAAGAGGGCGTGCT	pchF	PA4225	pyochelin synthetase
81	64	4736862	-	CTTGCCCGCCACTTGCA	pchF	PA4225	pyochelin synthetase
77	465	4741576	-	CTGGTCGGCGCCTTGCA	pchE	PA4226	dihydroaeruginoic acid synthetase

NC_002947.3 Pseudomonas putida KT2440, complete genome

1 NRPS 4767855-4798662

Score	Distance From ORF	N	Strand	Sequence	Gene	Synonym	Protein Name
79	278	4795256	-	TTGGCGCCGAAGGTGCA	-	PP_4222	syrP protein, putative
77	431	4795409	-	ATGGGGCAGCGGTTGCG	-	PP_4222	syrP protein, putative
80	413	4796855	-	ATGGGCCAGGCATTGCT	-	PP_4223	diaminobutyrate--2-oxoglutarate aminotransferase

2 NRPS 4818235-4832793

Score	Distance From ORF	N	Strand	Sequence	Gene	Synonym	Protein Name
76	122	4832915	-	TTGGCCAGTGGCCTGCT	-	PP_4245	siderophore biosynthesis protein, putative

NC_004578.1 Pseudomonas syringae pv. tomato str. DC3000, complete genome

1 NRPS 2307009-2356936

Score	Distance From ORF	N	Strand	Sequence	Gene	Synonym	Protein Name
81	315	2306694	+	ATGGAATTCACCTTGCT	-	PSPTO_2134	pyoverdine synthetase, thioesterase component
76	381	2322147	+	CTGGTCGGTCATTTGCA	-	PSPTO_2137	MbtH-like protein
78	274	2324978	-	CCGGCGCCGAAATTGCT	-	PSPTO_2139	cation ABC transporter, permease protein
80	491	2327813	-	CTGGCACAAGTGGCGCT	-	PSPTO_2143	hypothetical protein

Score	Distance From ORF	N	Strand	Sequence	Gene	Synonym	Protein Name
75	384	2330265	+	CTGGAAAACGACGTGCT	-	PSPTO_2147	pyoverdine sidechain peptide synthetase I, epsilon-Lys module
77	389	2333571	+	CCGACACACTTGTTGCT	-	PSPTO_2148	pyoverdine sidechain peptide synthetase II, D-Asp-L-Thr component

2 NRPS/PKS 2863432-2890634

Score	Distance From ORF	N	Strand	Sequence	Gene	Synonym	Protein Name
76	322	2868091	-	TTGGCGCGGGTGCCGCA	-	PSPTO_2593	multidrug resistance protein, AcrA/AcrE family
79	78	2872757	-	GTGGCCAGGCTCTGGCA	irp4	PSPTO_2598	yersiniabactin synthetase, thioesterase component
81	235	2873968	-	CTGGCGCAACACTCGCA	irp3	PSPTO_2599	yersiniabactin synthetase, thiazolinyl reductase component
80	112	2884576	-	CTGGCGCGGCACTTGAG	-	PSPTO_2601	hypothetical protein
78	384	2884848	-	CTGGCGCTGACGTGGCT	-	PSPTO_2601	hypothetical protein
75	89	2890723	-	CAGGCTCGGAACTGGCA	-	PSPTO_2602	yersiniabactin non-ribosomal peptide synthetase

3 NRPS 3151815-3185293

Score	Distance From ORF	N	Strand	Sequence	Gene	Synonym	Protein Name
76	472	3152407	+	TTGATACGGCTGATGCT	syfA	PSPTO_2829	non-ribosomal peptide synthetase SyfA

4 NRPS 5087432-5106501

Score	Distance From ORF	N	Strand	Sequence	Gene	Synonym	Protein Name
76	440	5088005	+	CTGGCAGCCTGGTTCCT	-	PSPTO_4518	non-ribosomal peptide synthetase, initiating component

5 PKS 5282597-5304804

Score	Distance From ORF	N	Strand	Sequence	Gene	Synonym	Protein Name
75	23	5284944	+	CTGGTCCGCAAGTCGCT	cfa3	PSPTO_4683	coronafacic acid beta-ketoacyl synthetase component
75	194	5286445	+	CTGCCAGGCCAACTGCT	cfa5	PSPTO_4685	coronafacic acid synthetase, ligase component

6 NRPS 5313602-5327197

NC_003198.1 Salmonella enterica subsp. enterica serovar Typhi str. CT18, complete genome

1 NRPS 624926-644794

Score	Distance From ORF	N	Strand	Sequence	Gene	Synonym	Protein Name
82	483	640548	-	CTGGCGCAATTTCTGCT	fepB	STY0638	iron-enterobactin transporter periplasmic binding protein
79	288	641267	+	CTGGCCTGTCTGCTGCA	entE	STY0640	enterobactin synthase subunit E

NC_004347.1 Shewanella oneidensis MR-1, complete genome

1 PKS 1670313-1686159

Score	Distance From ORF	N	Strand	Sequence	Gene	Synonym	Protein Name
78	441	1676645	-	ATAGCATGAGTATTGCC	-	SO_1599	beta keto-acyl synthase

NC_003143.1 Yersinia pestis CO92, complete genome

**1**	NRPS		839849-856936					
	Score	Distance From ORF	N	Strand	Sequence	Gene	Synonym	Protein Name
	79	186	841919	-	CGGGCACTATTGCTGCT	-	YPO0774	hypothetical protein
	81	353	857289	-	ATGGCCCCCCGCCTGCT	-	YPO0778	putative siderophore biosysnthesis protein
**2**	NRPS/PKS		2140840-2169669					
	Score	Distance From ORF	N	Strand	Sequence	Gene	Synonym	Protein Name
	82	239	2145615	-	CTGGCGCGACTGCTGCG	irp4	YPO1908	yersiniabactin biosynthetic protein YbtT
	75	486	2166571	+	ATGACACGCTGCTGGCG	irp8	YPO1915	putative signal transducer
	83	441	2167924	+	CTGGCCGGCGGATTGCT	ybtS	YPO1916	salicylate synthase Irp9
	79	96	2168269	+	GGGGCATTCACGTTGCT	ybtS	YPO1916	salicylate synthase Irp9
